# Epithelial MAPK signaling directs endothelial NRF2 signaling and IL-8 secretion in a tri-culture model of the alveolar-microvascular interface following diesel exhaust particulate (DEP) exposure

**DOI:** 10.1186/s12989-024-00576-8

**Published:** 2024-03-11

**Authors:** Eva C. M. Vitucci, Alysha E. Simmons, Elizabeth M. Martin, Shaun D. McCullough

**Affiliations:** 1 Interdisciplinary Faculty of Toxicology, School of Public Health, Texas A&M University, College Station, TX USA; 2 Curriculum in Toxicology, School of Medicine, University of North Carolina, Chapel Hill, NC USA; 3 The Center for Environmental Medicine, Asthma and Lung Biology, School of Medicine, University of North Carolina, Chapel Hill, NC USA; 4 Epigenetics and Stem Cell Biology Laboratory, National Institute of Environmental Health Sciences, Durham, NC USA; 5 Exposure and Protection, RTI International, 3040 East Cornwallis Road, Durham, NC USA; 6 Public Health and Integrated Toxicology Division, Center for Public Health and Environmental Assessment, U.S. Environmental Protection Agency, Chapel Hill, NC USA

**Keywords:** Alveolar epithelial cells, Microvascular endothelial cells, Transcriptomics, Redox dysfunction, Tri-culture models, PM_2.5_, NRF2 signaling, MAPK signaling

## Abstract

**Background:**

Particulate matter 2.5 (PM_2.5_) deposition in the lung’s alveolar capillary region (ACR) is significantly associated with respiratory disease development, yet the molecular mechanisms are not completely understood. Adverse responses that promote respiratory disease development involve orchestrated, intercellular signaling between multiple cell types within the ACR. We investigated the molecular mechanisms elicited in response to PM_2.5_ deposition in the ACR, in an in vitro model that enables intercellular communication between multiple resident cell types of the ACR.

**Methods:**

An in vitro, tri-culture model of the ACR, incorporating alveolar-like epithelial cells (NCI-H441), pulmonary fibroblasts (IMR90), and pulmonary microvascular endothelial cells (HULEC) was developed to investigate cell type-specific molecular responses to a PM_2.5_ exposure in an in-vivo-like model. This tri-culture in vitro model was termed the alveolar capillary region exposure (ACRE) model. Alveolar epithelial cells in the ACRE model were exposed to a suspension of diesel exhaust particulates (DEP) (20 µg/cm^2^) with an average diameter of 2.5 µm. Alveolar epithelial barrier formation, and transcriptional and protein expression alterations in the directly exposed alveolar epithelial and the underlying endothelial cells were investigated over a 24 h DEP exposure.

**Results:**

Alveolar epithelial barrier formation was not perturbed by the 24 h DEP exposure. Despite no alteration in barrier formation, we demonstrate that alveolar epithelial DEP exposure induces transcriptional and protein changes in both the alveolar epithelial cells and the underlying microvascular endothelial cells. Specifically, we show that the underlying microvascular endothelial cells develop redox dysfunction and increase proinflammatory cytokine secretion. Furthermore, we demonstrate that alveolar epithelial MAPK signaling modulates the activation of NRF2 and IL-8 secretion in the underlying microvascular endothelial cells.

**Conclusions:**

Endothelial redox dysfunction and increased proinflammatory cytokine secretion are two common events in respiratory disease development. These findings highlight new, cell-type specific roles of the alveolar epithelium and microvascular endothelium in the ACR in respiratory disease development following PM_2.5_ exposure. Ultimately, these data expand our current understanding of respiratory disease development following particle exposures and illustrate the utility of multicellular in vitro systems for investigating respiratory tract health.

**Supplementary Information:**

The online version contains supplementary material available at 10.1186/s12989-024-00576-8.

## Background

Respiratory diseases are among the leading causes of death in America and are an increasing, incurable burden [15, 48, 62, 96]. While underlying factors can cause respiratory diseases, exposure of the pulmonary epithelium to inhaled materials from occupational, behavioral, or environmental settings commonly drive their development [20, 32]. Particulate matter air pollution with an aerodynamic diameter of 2.5 µm and below (PM_2.5_) causes respiratory disease and is a major contributor to cardiopulmonary mortality worldwide. Due to its small size, PM_2.5_ deposits in the pulmonary region of the lung, which includes the alveolar-capillary interface region (ACR) consisting of the alveolar epithelium, interstitial fibroblasts, and capillary microvascular endothelial cells. Many studies have leveraged in vitro monoculture systems to examine the effect of PM_2.5_ and the underlying mechanisms on the respiratory tract; however, due to the limited biological complexity of monoculture models, the cellular and molecular mechanisms responsible for the exposure effects in the ACR remain poorly understood.

The alveolar epithelium lines approximately 480 million alveoli in the average adult male [28, 64, 95], making it the largest epithelial surface area in the human body. The capillaries that are immediately beyond this epithelial surface area are composed of microvascular endothelial cells and enable the exchange of gases, such as oxygen and carbon dioxide, between inhaled air and circulating blood across the alveolar epithelial-capillary endothelial barrier [28, 37, 64, 95]. PM_2.5_ deposition in the ACR induces the accumulation of reactive oxygen oxidative species (ROS), redox dysfunction, and activates pulmonary inflammatory signaling [19, 32, 46]. These adverse responses involve orchestrated cell signaling between the alveolar epithelium and the surrounding capillary endothelium,however, the mechanisms involved are not well characterized [9, 10, 30, 33, 92]. Thus, investigating the intercellular signaling mechanisms between the alveolar epithelium and endothelium following PM_2.5_ deposition is a viable approach to improve our understanding of PM_2.5_ -induced respiratory disease development in the ACR. To improve our mechanistic understanding of exposure responses in the ACR, we developed a tri-culture in vitro model of the ACR. This alveolar capillary region exposure (ACRE) model includes alveolar epithelial cells, interstitial lung fibroblasts, and lung microvascular endothelial cells that are assembled to resemble the architecture of the ACR in vivo. Further, the model was designed to allow the separation of each cell type for analysis to investigate cell-type specific biology and responses to inhalable material exposures and exogenous stimuli.

Diesel exhaust particulates (DEP), a ubiquitous component of PM_2.5_, cause pulmonary redox dysfunction, inflammation, and respiratory disease development [50, 54, 74, 84]. Here, we used the ACRE model to examine the cellular and molecular mechanisms involved in the effects of the model oxidant and inhalable particulate, DEP, on the ACR. We observed that exposure of the alveolar epithelium in the ACRE model to DEP (ACRE-DEP exposure) induces a robust transcriptional response in the underlying microvascular endothelium. Further, we identified that an ACRE-DEP exposure induces nuclear factor erythroid 2 factor 2 (NRF2)-dependent secretion of the pro-inflammatory cytokine, interleukin (IL)-8 from endothelial cells. Both the increased endothelial NRF2 protein stabilization and IL-8 secretion were dependent on the DEP-induced activation of the mitogen activated protein kinase (MAPK), extracellular signaling related kinases 1/2 (ERK1/2), in the exposed alveolar epithelial cells. These data suggest that the microvascular endothelium plays an important role in the secretion of pro-inflammatory mediators following epithelial exposure to inhaled materials and epithelial signaling. Our findings also illustrate that distinct, intracellular signaling pathways in the alveolar epithelium and microvascular endothelium contribute synergistically to this increased secretion of pro-inflammatory mediators. Together, we report novel mechanisms of the ACR response to DEP exposure and demonstrate how tri-culture models, such as the ACRE model, can improve our understanding of the pathogenic mechanisms promoting respiratory disease development following exposure to inhaled materials.

## Results

### The ACRE model exhibits key features of the alveolar-capillary interface

**Fig. 1:**
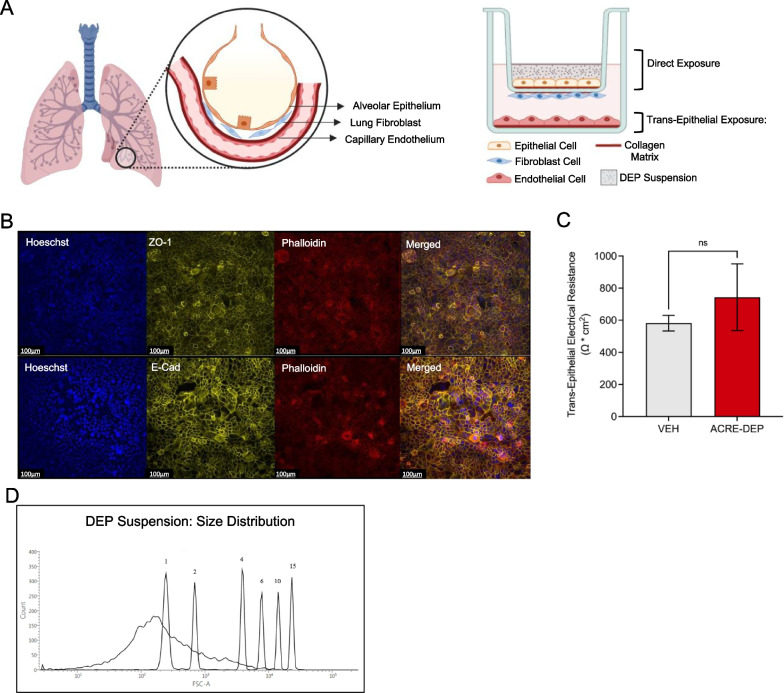
ACRE model exhibits epithelial barrier function. **A** Illustrated representation of the ACR in vivo and of the alveolar capillary region exposure (ACRE) model in vitro. During an ACRE-DEP exposure, alveolar-like epithelial cells are directly exposed to DEP suspension. In contrast, microvascular lung endothelial cells are never in direct contact with DEP. **B** Representative epithelial immunofluorescent images of ZO-1 and E-cadherin tight and adherens junctions, respectively, that colocalized with phalloidin-stained actin after a 24 h ACRE-DEP exposure. Images were acquired using a 20X objective. Scale bar represents 100 µm. Representative image of n = 3 independent experiments is shown. **C** TEER across the epithelial monolayer after a 24 h VEH or 24 h ACRE-DEP exposure. Values represent the mean of n = 3 independent experiments ± SD. **D** Distribution of DEP agglomerate diameter. The diameter of DEP agglomerates after resuspension by sonication was measured relative to the size standards included in the Flow Cytometry Size Calibration Kit (1, 2, 4, 6, 10, and 15 µm)

The formation of an alveolar epithelial barrier is essential for a functional alveolar epithelium in vivo [9, 10, 30]. Alveolar epithelial barrier formation is characterized by the formation of tight and adherens junctions, high transepithelial electrical resistance (TEER), and low transepithelial permeability of small molecular weight compounds [27, 71, 76, 82]. Immunofluorescent staining of the epithelial layer of the ACRE model exhibited co-localization of phalloidin-stained actin filaments with the tight junction forming protein, ZO-1, and the adherens junction forming protein, E-cadherin, at cell–cell junctions (Fig. 1A, B). Colocalization of these proteins is consistent with the formation of an intact epithelial cell barrier and was not perturbed by the addition of vehicle (VEH) or by 24 h ACRE-DEP exposures. Structural evidence of epithelial barrier formation was supported by TEER values reflective of an intact epithelial barrier [71, 82] following a 24 h VEH and 24 h ACRE-DEP exposure (582 ± 28 Ω × cm^2^ and 743 ± 120 Ω × cm^2^, respectively) (Fig. 1C). There was no significant difference in TEER between 24 h VEH and 24 h ACRE-DEP exposure. These data support that a 24 h ACRE-DEP exposure does not disrupt the alveolar epithelial barrier in the ACRE model. As epithelial barrier formation in vivo can be influenced by the viability of the tissue [25], we investigated the cytotoxic effects of DEP in the ACRE model. There was no significant induction or difference in cytotoxicity between the 24 h VEH and 24 h ACRE-DEP in the ACRE model (Additional File 3: Figure S1). Together, these data support the presence of a functional alveolar epithelial barrier and present a tri-culture model of the ACR to investigate the molecular responses of DEP in the absence of cytotoxicity.

### Lung alveolar epithelial cells and microvascular endothelial cells have distinct transcriptional responses to ACRE-DEP exposure in the ACRE model

**Fig. 2:**
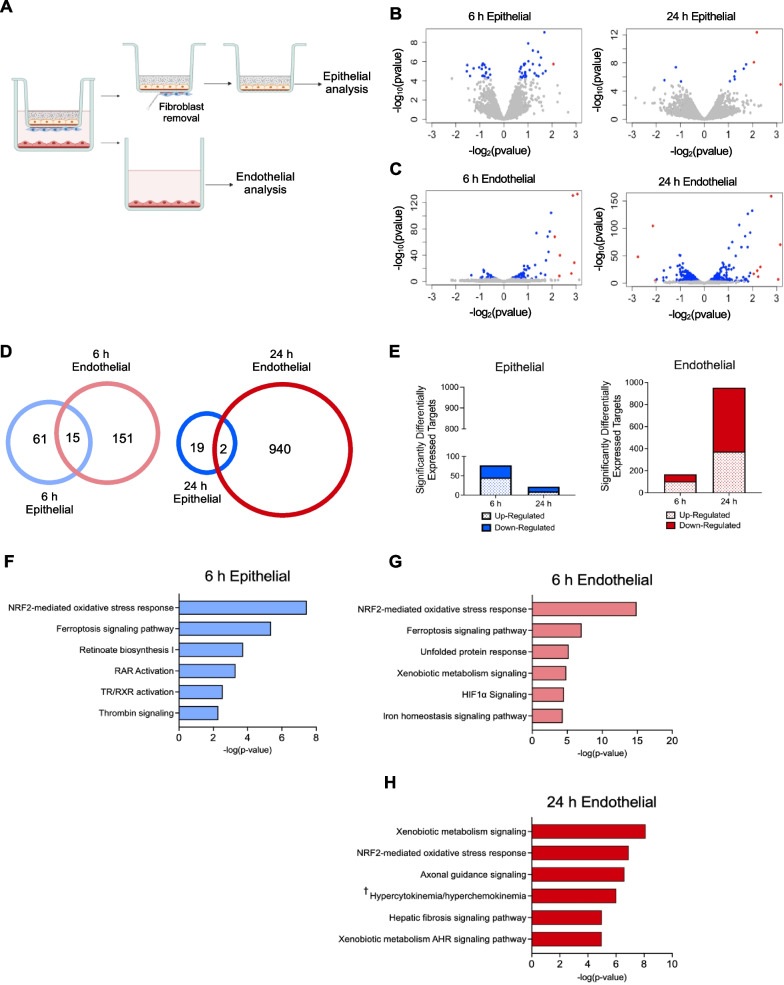
Microvascular endothelial cells have a large and distinct transcriptional response to an ACRE-DEP exposure. RNA-sequencing was performed on the epithelial and endothelial cells after a 6 h and 24 h VEH and ACRE-DEP exposure. **A** Illustrated representation of the separation of the epithelial and endothelial cells from the ACRE model and removal of the fibroblast layer following an ACRE-DEP exposure. **B**, **C** Volcano plots of the differentially expressed genes in the **B** epithelial cells and **C** endothelial cells after a 6 h and 24 h ACRE-DEP exposure. The x-axis shows the log_2_ fold change of differentially expressed targets in the epithelial and endothelial cells after an ACRE-DEP exposure relative to VEH. The y-axis shows the negative log_10_ of the two-tailed test *P* value. Blue dots represent targets with an adjusted *p*-value of *p* $\le$ 0.1. Red dots represent targets with an adjusted *p*-value of *p* $\le$ 0.1 and a log2fold change > 2. **D** Venn diagrams, sorted by exposure length, of the differentially expressed genes in the epithelial and endothelial cells. **E** Gene counts of significantly, differentially expressed genes in the epithelial and the endothelial cells. **F**–**H** IPA of the top predicted, significantly activated pathways in the **F** epithelial and endothelial cells after a 6 h (**G**) and 24 h ACRE-DEP (**H**) exposure. **B**–**H** Represent n = 3 independent experiments. † = Role of hypercytokinemia/hyperchemokinemia in the pathogenesis of influenza

In addition to serving as a functional barrier between the respiratory and cardiovascular systems, the alveolar epithelium and microvascular endothelium play important roles in mediating the response to inhaled materials and the development of respiratory disease [35, 41, 50, 60, 84]. To identify the cell-type specific mechanisms elicited in the ACR in response to DEP, we performed RNA-sequencing of the epithelial and the endothelial cells following a 6 h and 24 h ACRE-DEP exposure (Fig. 2A). We observed distinct transcriptional responses in the epithelial and endothelial compartments (Fig. 2B–E). There were 76 and 21 differentially expressed genes in the epithelium following a 6 h and 24 h ACRE-DEP exposure, respectively (Fig. 2D–E). Despite the endothelial cells being separated from the exposure material by the alveolar epithelial barrier and underlying fibroblast monolayer, there were 166 and 942 differentially expressed genes in the endothelium following a 6 h and 24 h ACRE-DEP exposure, respectively (Fig. 2D, E). There were only 15 and 2 genes alternatively regulated in both the epithelial and endothelial cells following a 6 h and 24 h ACRE-DEP exposure, respectively (Fig. 2D). Moreover, while the epithelial transcriptional response decreased over the 24 h exposure, the endothelial transcriptional response substantially increased (Fig. 2E). These data illustrate distinct, cell-type specific transcriptional responses, and emphasize the robust transcriptional response in the microvascular endothelium following an ACRE-DEP exposure.

To identify the top canonical pathways activated in each cell type, we assessed differentially expressed gene profiles by Ingenuity Pathway Analysis (IPA). The top 2 canonical pathways enriched in both the epithelial and endothelial cells after a 6 h ACRE-DEP exposure were the NRF2-mediated oxidative stress response and ferroptosis signaling pathway, with both pathways being more significantly enriched in the endothelial cells (Figs. 2F, G and Additional file 1: Table S1). The remaining 4 of the top 6 canonical pathways activated in the epithelial and endothelial following a 6 h ACRE-DEP exposure were unique to each of the two cell types. Following a 6 h ACRE-DEP exposure, lung development related pathways were enriched in the epithelial cells (Fig. 2F) and xenobiotic metabolism and hypoxia signaling related pathways were enriched in the endothelial cells (Fig. 2G). Following a 24 h ACRE-DEP exposure, there were no enriched canonical pathways that met selection criteria in the epithelial cells; however, analysis of the endothelial cells following a 24 h ACRE-DEP exposure again identified oxidative stress response and xenobiotic metabolism related pathways as the top significantly alternatively regulated pathways (Fig. 2H). This persistent detection of perturbed canonical pathways in only the microvascular endothelial cells further supports cell-type specific transcriptional responses to an ACRE-DEP exposure.

### ACRE-DEP exposure induces alveolar epithelial and microvascular endothelial antioxidant expression

**Fig. 3:**
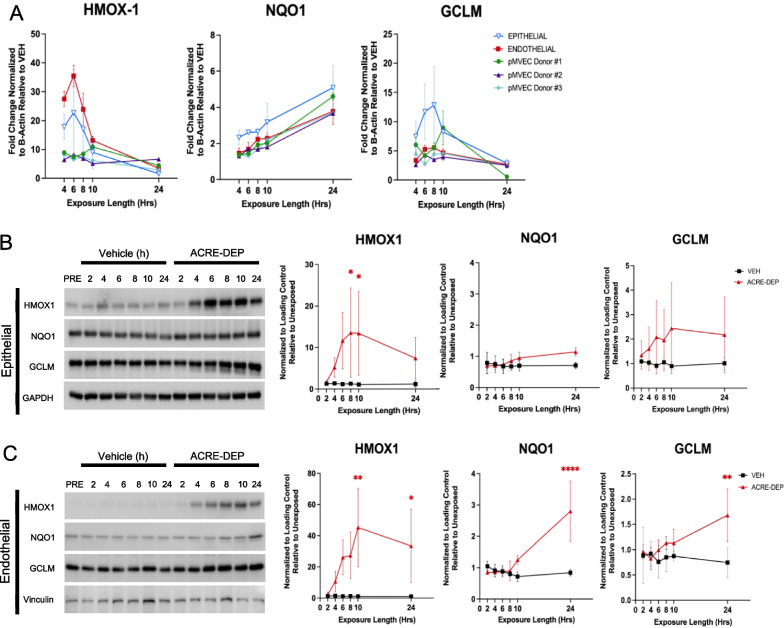
ACRE-DEP induces alveolar epithelial and microvascular endothelial antioxidant expression. **A** mRNA expression, determined by qPCR, of *HMOX1*, *NQO1*, and *GCLM* in epithelial, endothelial, and pMVEC donors over a 4–24 h ACRE-DEP exposure. **B**, **C** Protein expression and densitometry of HMO1, NQO1, GCLM, and GAPDH or vinculin (loading controls) in the **B** epithelial cells and **C** endothelial cells over a 2–24 h ACRE-DEP exposure. **A**–**C** Values represent the mean of n = 3 independent experiments ± SD and immunoblots are representative images from n = 3 independent experiments. Statistically significant differences between vehicle and ACRE-DEP treated cells are indicated by *p ≤ 0.05, **p ≤ 0.01, and ****p ≤ 0.0001

Analysis of the global transcriptional response following a 6 h and 24 h ACRE-DEP exposure indicated that oxidative stress responsive pathways were significantly alternatively regulated in the alveolar epithelial (6 h) and the endothelial cells (6 and 24 h) (Fig. 2F, H). The induction of genes such as heme oxygenase 1 (HMOX1), NAD(P)H quinone dehydrogenase 1 (NQO1), and glutamate-cysteine ligase modifier (GCLM) often occurs as a part of the oxidative stress response following exposures to inhaled toxicants [29, 53, 75, 100]. Therefore, to validate the activation and investigate the cell-type specific kinetics of the ACRE-DEP-induced oxidative stress response, we evaluated the transcript levels, using quantitative PCR (qPCR), of these antioxidants over a 4–24 h time course in the epithelial and endothelial cells in the ACRE system. ACRE-DEP exposure induced *HMOX1* transcript in both cell types at all timepoints evaluated with peak fold change induction of 22.7 ± 14.7 and 35.4 ± 3.7 occurring at 6 h in epithelial and endothelial cells, respectively (Fig. 3A). In contrast to *HMOX1*, *NQO1* peak transcript induction of 5.1 ± 1.2 and 3.8 ± 0.7 occurred in the epithelial and endothelial cells, respectively, following a 24 h ACRE-DEP exposure. *GCLM* peak transcript induction of 12.8 ± 6.5 and 5.5 ± 0.4 occurred in the epithelial and endothelial cells, respectively, following an 8 h ACRE-DEP exposure. Observations in the HULEC cell line were validated by qPCR using primary lung microvascular endothelial cells from 3 healthy donors. These donors exhibited elevated *HMOX1, NQO1,* and *GCLM* induction, with similar kinetic profiles as the HULEC (Fig. 3A). The induction of these genes at the protein level was subsequently confirmed by time-course immunoblotting in both cell types over a 2–24 h ACRE-DEP exposure. In the epithelium, only HMOX1 protein levels were significantly increased after 8 h and 10 h of exposure, with peak fold change induction of 13.6 ± 10.7 and 13.4 ± 10.1, respectively (Fig. 3B). In the endothelium, HMOX1, NQO1, and GCLM protein levels were significantly increased and followed a similar expression pattern as their respective transcripts. Specifically, HMOX1 protein was significantly elevated after 10 h and 24 h of exposure (peak fold change induction of 45.3 ± 25.2 and 33.5 ± 23.4, respectively), and NQO1 and GCLM were significantly elevated after 24 h of exposure (peak fold change induction of 2.8 ± 0.9 and 1.7 ± 0.5, respectively) (Fig. 3C). Together, these findings reveal cell type specific oxidative stress responses and suggest a more robust response in the microvascular endothelium.

### ACRE-DEP induces alveolar epithelial MAPK signaling and microvascular endothelial NRF2 signaling

**Fig. 4:**
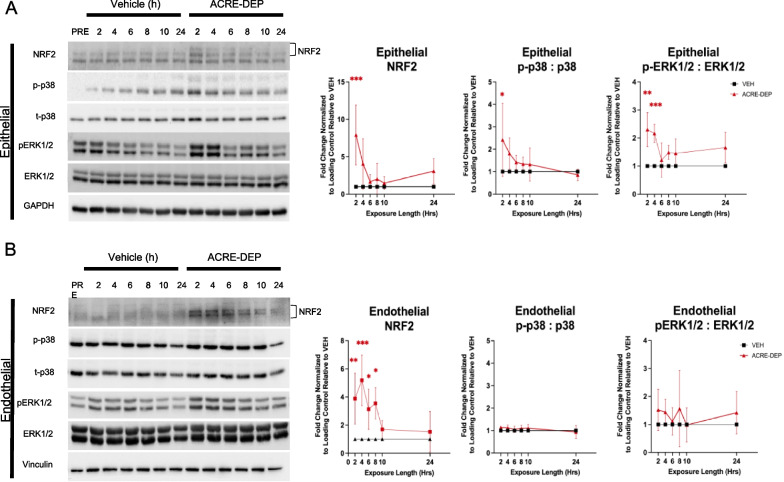
ACRE-DEP induces alveolar epithelial MAPK signaling and microvascular endothelial NRF2 signaling. **A**, **B** Protein expression and densitometry of NRF2, phosphorylated and total ERK1/2, phosphorylated and total p38, and GAPDH or vinculin (loading controls) in **A** epithelial cells and **B** endothelial cells over a 2 – 24 h ACRE-DEP exposure. **A**, **B** Values represent the mean of n = 3 independent experiments ± SD and immunoblots are representative images from n = 3 independent experiments. Statistically significant differences between vehicle and ACRE-DEP treated cells are indicated by *p ≤ 0.05, **p ≤ 0.01, and ***p ≤ 0.001

The NRF2, ERK1/2 and p38 MAPK, and nuclear factor kappa B (NF-κB) signaling pathways are commonly involved in mediating oxidative stress responses and antioxidant gene transcription [73, 79, 80, 94]. Antioxidant gene transcription was induced in epithelial and endothelial cells as early as 4 h of ACRE-DEP exposure. To investigate whether these signaling pathways were potential mediators of this antioxidant expression, we evaluated the activation of NRF2, ERK1/2, p38, and NF-κB pathways by immunoblotting over a 2–24 h ACRE-DEP exposure time course. While levels of NRF2 protein increased 7.9 ± 4.0-fold in epithelial cells at 2 h, NRF2 protein levels were elevated in endothelial cells with significant increases of 3.9 ± 1.8, 5.2 ± 1.8, 3.1 ± 1.4, and 3.5 ± 1.1-fold occurring between 2 and 8 h (Fig. 4A, B). Levels of phosphorylated p38 were elevated 2.4 ± 1.6-fold in epithelial cells at 2 h, while ERK1/2 phosphorylation was elevated at 2 h and 4 h (2.3 ± 0.6 and 2.2 ± 0.3-fold, respectively) (Fig. 4A). We did not observe any significant effects of ACRE-DEP exposure on p65 phosphorylation at any time point in either cell type (Additional File 3: Figures S2A-S2B. ROS can induce NRF2 stabilization and activation, thus we evaluated whether ACRE-DEP induced endothelial ROS accumulation. We detected a significant increase in ROS accumulation over a 24 h ACRE-DEP exposure compared to a 24 h VEH exposure (7383.96 ± 642.42 and 4668.22 ± 438.3 arbitrary CellRox fluorescent units, respectively; Additional File 3: Figure S3A) (CellRox procedure described in Additional File 5: Method S1). This increase in endothelial ROS accumulation, despite the return of endothelial NRF2 protein levels to baseline, suggests an ACRE-DEP exposure induces endothelial redox dysfunction.

### Microvascular endothelial antioxidant expression is NRF2 dependent

**Fig. 5:**
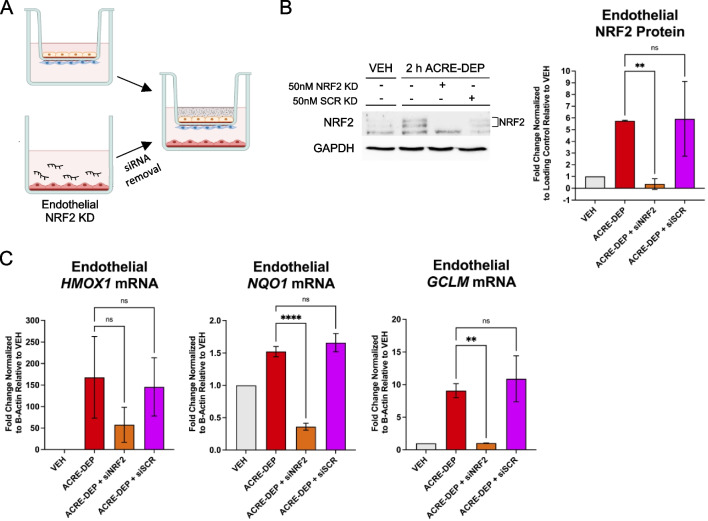
Microvascular endothelial antioxidant gene expression is NRF2 dependent. **A** Illustrated representation of endothelial NRF2 knock down using small interfering RNA (siNRF2). **B** Protein expression and densitometry of NRF2 stabilization and GAPDH (loading control) in endothelial siNRF2 cells after a 2 h ACRE-DEP exposure. **C** mRNA expression of the antioxidants *HMOX1*, *NQO1*, and *GCLM* in endothelial siNRF2 cells after a 6 h ACRE-DEP exposure. **B**, **C** Values represent the mean of n = 3 independent experiments ± SD and immunoblots are representative images from n = 3 independent experiments. Statistically significant differences between ACRE-DEP and ACRE-DEP + siNRF2 cells are indicated by **p ≤ 0.01 and ****p ≤ 0.0001

The NRF2 transcription factor regulates *HMOX1*, *NQO1*, and *GCLM* gene expression [53, 75]. To determine if the accumulation of endothelial NRF2 was required for the ACRE-DEP mediated induction of *HMOX1*, *NQO1*, and *GCLM* we knocked down NRF2 in the endothelial cells using a pool of NRF2 targeting small interfering RNAs (siRNA) (Fig. 5A). Targeted treatment of endothelial cells with NRF2 siRNA significantly decreased *NRF2* protein and mRNA levels following ACRE-DEP exposure compared to siScramble (siSCR) controls (Fig. 5B and Additional file 2: Table S2). NRF2 siRNA knockdown significantly decreased transcript levels of *HMOX1*, *NQO1*, and *GCLM*, relative to siScramble treatment, following an ACRE-DEP exposure in endothelial cells (Fig. 5C). These findings suggest endothelial expression of *HMOX1*, *NQO1*, and *GCLM* is NRF2 driven.

### Alveolar epithelial MAPK signaling activates microvascular endothelial NRF2-dependent antioxidant gene expression

**Fig. 6:**
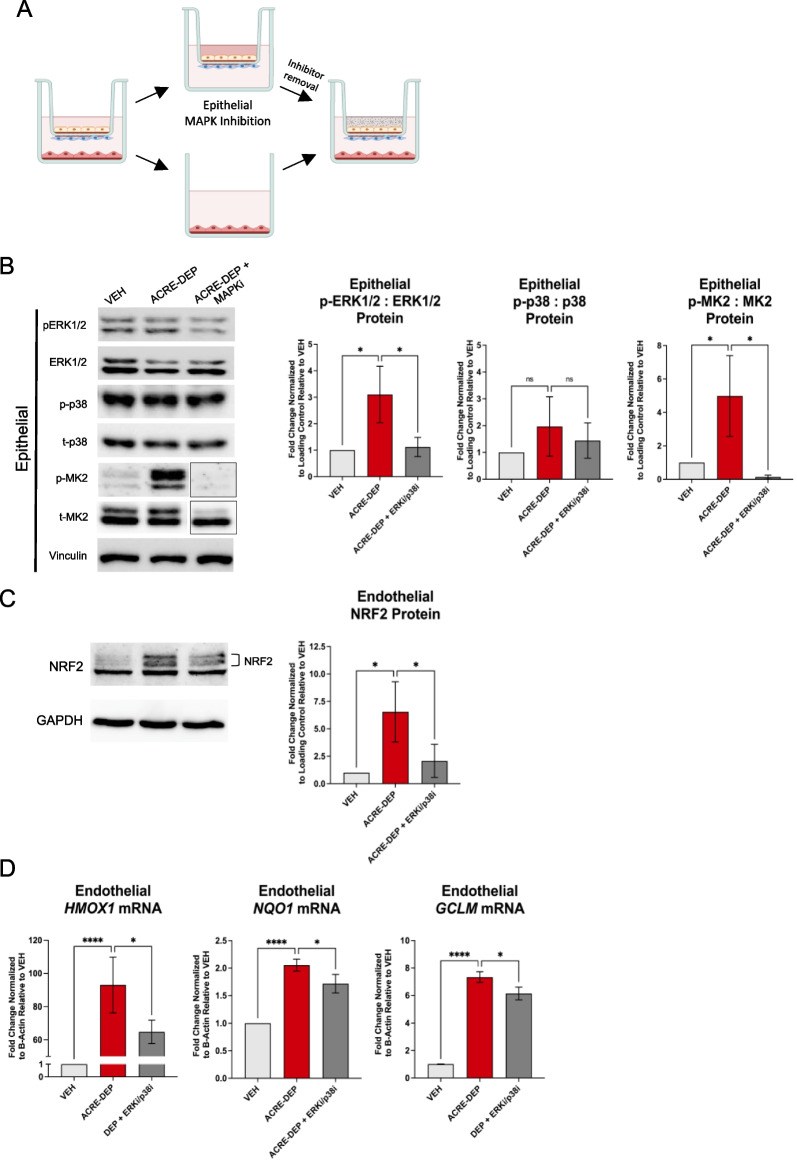
Alveolar epithelial MAPK signaling activates microvascular endothelial NRF2-dependent antioxidant gene expression. **A** Illustrated representation of epithelial cell MAPK inhibition pre-treatment with the ERK1/2 inhibitor, SCH772984, and the p38 inhibitor, LY2228820 (ERKi/p38i). **B** Protein expression and densitometry of phosphorylated and total ERK1/2, p38, MK2, and vinculin (loading control), in epithelial cells after a 1 h ACRE-DEP exposure. Note: the outlined p-MK2 and t-MK2 blots are from the same immunoblot as the shown VEH and DEP bands; however, they were not originally ordered in the same pattern as the other samples in **B**, thus the bands outlined with the black border were re-ordered as shown. **C** Protein expression and densitometry of NRF2 stabilization and GAPDH (loading control) in endothelial cells after a 2 h ACRE-DEP exposure. **D** mRNA expression of HMOX1, NQO1, and GCLM in endothelial cells after a 6 h ACRE-DEP exposure. **B**–**D** Values represent the mean of n = 3 independent experiments ± SD and immunoblots are representative images from n = 3 independent experiments. Statistically significant differences between VEH, ACRE-DEP, and DEP + ERKi/p38i cells are indicated by *p ≤ .05 and ****p ≤ .0001

ERK1/2 and p38 phosphorylation was significantly elevated in epithelial cells following 2 and 4 h ACRE-DEP exposure. ERK1/2 and p38 modulate intracellular NRF2 activation, yet it is unclear if these MAPKs also regulate intercellular NRF2 activation [52, 78, 101]. To identify the role epithelial MAPK intercellular signaling plays in modulating the observed endothelial NRF2 activation, we temporarily separated Transwell^®^ inserts from the ACRE model to allow for epithelial-only pre-treatment with SCH772984 and LY2228820, inhibitors of ERK1/2 and p38 activity, respectively. Following the pre-treatment, the inhibitors were removed, and the ACRE model was reassembled for an ACRE-DEP exposure (Fig. 6A). Pre-treatment of the epithelial compartment with the combination of ERK1/2 and p38 inhibitors significantly decreased MAPK activity as indicated by the significant decrease in phosphorylation of ERK1/2 and phosphorylation of mitogen activated protein kinase-activated protein kinase 2 (MK2), the downstream target of p38, in the epithelial cells induced by ACRE-DEP exposure (Fig. 6B). In addition to direct effects on the epithelial compartment, the combined inhibitor pre-treatment significantly decreased NRF2 protein stabilization caused by ACRE-DEP exposure in endothelial cells (Fig. 6C). Similar to the effect of NRF2 siRNA knockdown in endothelial cells, treatment of epithelial cells with MAPK inhibitors significantly decreased the induction of HMOX1, NQO1, and GCLM transcripts in adjacent endothelial cells (Fig. 6D). Verification experiments using the different ERK1/2 and p38 inhibitors, BVD-523 and SB203580, respectively, demonstrated significant decreases in the abundance of HMOX1 transcript in the endothelial cells following ACRE-DEP exposure (Additional file 3: Figure S4A). These results indicate that the ACRE-DEP induced epithelial MAPK signaling directs the observed endothelial NRF2 signaling and subsequent antioxidant gene expression.

### Alveolar epithelial MAPK signaling modulates microvascular endothelial NRF2-dependent IL-8 expression and secretion

**Fig. 7:**
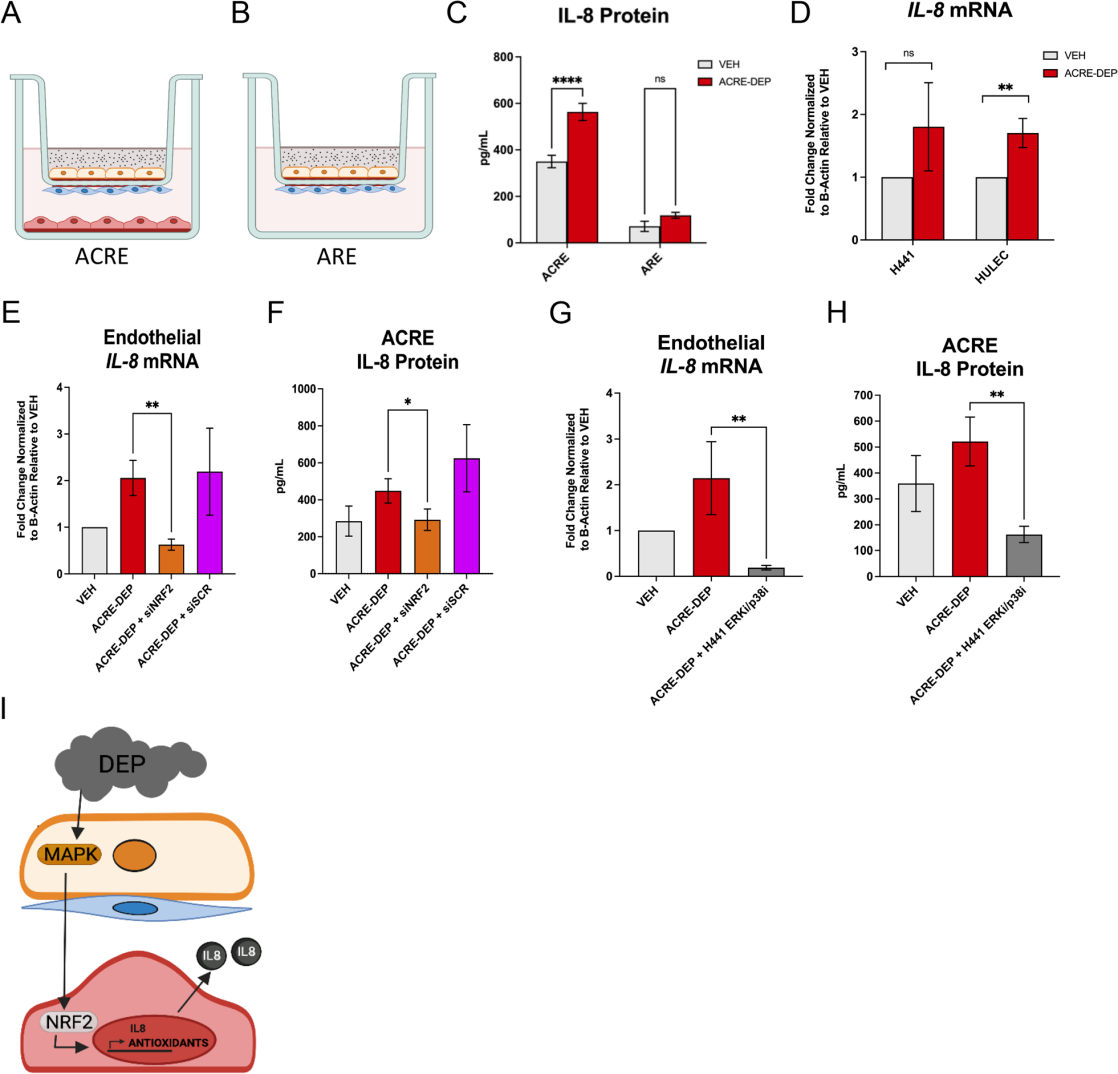
Alveolar epithelial MAPK signaling modulates microvascular endothelial NRF2-dependent IL-8 expression and secretion. Illustrated representative image of the **A** ACRE model and **B** the Alveolar Region Exposure (ARE) model in the absence of endothelial cells following an ACRE-DEP exposure. **C** IL-8 secretion in the basolateral medium of the ACRE and ARE model after a 6 h ACRE-DEP exposure. **D** mRNA expression of *IL-8* in the microvascular endothelial cells and alveolar epithelial cells after a 6 h ACRE-DEP exposure. **E** mRNA expression of *IL-8* in the NRF2 KD endothelial cells after a 6 h ACRE-DEP exposure. **F** IL-8 secretion in the basolateral medium of the ACRE model after a 6 h ACRE-DEP exposure with endothelial siNRF2 cells. **G** mRNA expression of *IL-8* in endothelial cells after a 6 h ACRE-DEP exposure with pre-treated, MAPK inhibited, epithelial cells (DEP + ERKi/p38i). **H** IL-8 secretion in the basolateral medium of the ACRE model after a 6 h ACRE-DEP exposure with MAPK inhibited epithelial cells (DEP + ERKi/p38i). **I** Summary illustration. **C**–**H** Values represent the mean of n = 3 independent experiments ± SD. Statistically significant differences between VEH, ACRE-DEP, siNRF2, and DEP + ERKi/p38i cells are indicated by *p ≤ 0.05, **p ≤ 0.01, and ****p ≤ 0.0001

Epithelial MAPK signaling regulates the expression of pro-inflammatory cytokines in response to a variety of inhalation insults [11, 69, 70, 93, 98]. While previous in vitro monoculture studies have investigated MAPK cytokine regulation in epithelial cells, multiple cell types within the lung can secrete pro-inflammatory cytokines in response to inhaled insult. Thus, we sought to determine whether the secretion of the pro-inflammatory cytokines IL-1$\beta$, TNF-$\alpha$, IL-6, and IL-8, which are frequently elevated following inhalation exposures, differed between the complete ACRE model (Fig. 7A) and the alveolar compartment (alveolar epithelium and fibroblasts) (Fig. 7B) following an ACRE-DEP exposure. Following an ACRE-DEP exposure, we only observed a significant increase in secretion of IL-8 and IL-1$\beta$ (Fig. 7C and Additional file 3: Figure S5A) in the complete ACRE model, and no significant pro-inflammatory cytokine secretion from the alveolar compartment alone (Fig. 7C and Additional file 3: Figure S5A). This increased level of IL-8 in the ACRE system corresponded to a significant increase in ACRE-DEP-induced *IL-8* transcript in endothelial, but not epithelial, cells (Fig. 7D). IL-8 production is responsive to changes in intracellular redox potential, redox dysfunction, and NRF2 signaling [57, 97, 99]. To determine whether endothelial NRF2 activation regulated *IL-8* expression and secretion, we knocked down NRF2 in the endothelial cells using siRNA and evaluated *IL-8* transcript levels and secreted protein levels in the basolateral conditioned medium. Endothelial NRF2 knockdown significantly attenuated ACRE-DEP-induced endothelial *IL-8* transcript levels and IL-8 protein levels in basolateral conditioned medium (Fig. 7E,F). In line with our earlier findings that epithelial MAPK signaling directs endothelial NRF2 activation, combined ERK and p38 inhibitor pre-treatment of the epithelial cells also resulted in a significant decrease in ACRE-DEP-induced endothelial *IL-8* transcript levels and protein levels in the basolateral conditioned medium (Fig. 7G,H). Combined pre-treatment using the different, second set of MAPK inhibitors resulted in a similar, significant decrease in endothelial *IL-8* levels following an ACRE-DEP exposure (Additional file 3: Figure S4B).

## Discussion

The inhalation of materials, such as PM_2.5_, initiates pathological processes that promote the development and/or exacerbation of respiratory diseases [20, 24, 50]. In vitro studies investigating the cellular and molecular mechanisms driving these adverse responses have primarily focused on the roles of the lung epithelium and resident immune cells using monoculture models. Using an in vitro, tri-culture model of the ACR, we show that the alveolar epithelium and capillary endothelium play distinct, but concerted, roles in the adverse exposure responses to the inhaled oxidative insult, DEP. Specifically, we demonstrate that alveolar epithelial MAPK signaling mediates microvascular endothelial NRF2-dependent IL-8 secretion following DEP exposure (Fig. 7I. These findings are significant because they (1 indicate that exposure-induced redox and pro-inflammatory signaling responses are not limited to the directly exposed pulmonary epithelial cells; (2 demonstrate that cells beyond the alveolar epithelial layer mediate exposure responses to inhaled insults; and (3 illustrate that exposure responses are dependent on distinct, but coordinated, cellular signaling pathways in adjacent cell types.

Exposure to DEP alters gene expression in respiratory epithelial cells [6, 27, 50, 84]. Consistent with previous studies, we observed DEP-induced changes in gene expression in epithelial cells in the ACRE model. Despite being separated from the exposure material by a functional alveolar epithelial barrier and fibroblast monolayer, we also observed a greater number of alternatively regulated genes in the microvascular endothelial cells following an ACRE-DEP exposure (Fig. 2B–E). Nearly all alternatively regulated gene expression in the epithelial cells had resolved by 24 h of the ACRE-DEP exposure. In contrast, the number of alternatively regulated genes in the endothelial cells was greatest by 24 h of the ACRE-DEP exposure (Fig. 2B–E). These findings support recent studies demonstrating that cells beyond the epithelial layer are more responsive to inhaled oxidative insults than the directly exposed epithelium [27]. Moreover, as Faber et al. [26] demonstrated a bronchial epithelial exposure can have exposure effects in an underlying fibroblast layer, these data demonstrate alveolar epithelial exposure effects can extend beyond this fibroblast layer, into a third cell layer, the endothelium. Future studies building off these findings to investigate the extent of inhalation exposure effects on cell types beneath the epithelial barrier in vitro will improve our understanding of the mechanisms driving the adverse local and systemic effects of these exposures. To our knowledge, this is the first comparison of the effects of an inhaled toxicant on the transcriptional profiles of alveolar epithelial and microvascular endothelial cells within a tri-culture, in vitro system of the ACR.

The induction of antioxidants following deposition of DEP, and other forms of PM_2.5_, on the directly exposed pulmonary epithelial layer is a common observation [2, 6, 22, 27, 50, 84, 100]. This induction is believed to be in response to the ROS, aromatic hydrocarbons, and metal ion co-contaminants released from the particles that catalyze intracellular ROS formation in the directly exposed cell layer [50, 84]. Here we show that the microvascular endothelium also increases antioxidant transcription throughout the ACRE-DEP exposure (Fig. 3A). In addition, despite the activation of NRF2 and antioxidant expression, we detected ROS accumulation in the microvascular endothelial cells following a 24 h ACRE-DEP exposure (Additional file 3: Figure S3A). These data indicate that the oxidative insult of DEP extends beyond the epithelial layer and induces redox dysfunction in the endothelium. In addition, the prolonged NRF2 signaling and antioxidant protein expression in the endothelium suggests the lung microvascular endothelium cannot resolve oxidative insults as efficiently as the alveolar epithelium (Fig. 3B, C). Thus, the microvascular endothelium may be more susceptible to injury and dysfunction. In support of this, clinical studies have observed markers of persistent endothelial dysfunction, such as impaired endothelial vasodilation, up to 24 h following a 1 h exposure to DEP [5, 51, 89]. Prolonged endothelial oxidative stress and dysfunction can have significant, adverse responses such as excessive pulmonary and systemic pro-inflammatory responses, thrombus formation, and the progression of atherosclerosis [14, 36, 38]. Therefore, inefficient endothelial intracellular stress resolution may be a key mechanism of how inhaled materials promote the development of respiratory and systemic diseases. Future studies using the ACRE model can investigate the differences in redox sensitivity between the alveolar epithelium and microvascular endothelium, the pulmonary exposure dosage required for endothelial dysfunction, and the mechanisms of this persistent endothelial dysfunction to help minimize the development of these diseases.

Direct exposure studies utilizing pulmonary epithelial and non-pulmonary endothelial monocultures demonstrate that redox sensitive signaling pathways, NF-κB, MAPK, and NRF2, regulate the transcription of redox sensitive targets following exposure to inhaled toxicants [4, 11, 18, 47, 55, 91, 93]. In addition, direct exposure of respiratory tract epithelial cells in vitro causes increased pro-inflammatory cytokine secretion [7, 11, 23, 83]. Thus, the respiratory epithelium has been depicted as the primary producer of pro-inflammatory signaling and mediator of adverse inflammatory responses after inhaled toxicant exposure. Consistent with these studies, DEP exposure of the alveolar epithelial cells in the ACRE model also activated the epithelial NRF2 and MAPK signaling pathways, ERK1/2 and p38 (Fig. 4A). In contrast, we did not observe activation of the NF-κB pathway in either cell type (Additional file 3: Figures S2A–S2B) nor a significant increase in epithelial secretion of pro-inflammatory cytokines (Fig. 7C and Additional file 3: Figure S5A). Instead, we identified a significant increase in IL-1 $\beta$ secretion and NRF2-dependent *IL-8* expression and secretion from the microvascular endothelial cells. In line with our finding, Teijaro et al. also observed increased endothelial inflammatory signaling and an amplified pro-inflammatory cytokine response, following pulmonary influenza viral infection, that contributes to the pathological, cytokine storm response [88]. Together, these findings implicate the microvascular endothelium as a mediator of inflammatory signaling after an inhaled toxicant exposure.

The secretion of pro-inflammatory cytokines, such as IL-8 and IL-1$\beta$, is positively correlated with respiratory disease development and pathogenesis [16, 65, 66]. Therapeutic strategies targeting these cytokines and their receptors have led to significant improvements in patient outcomes, further supporting their role in disease pathogenesis [16, 66]. PM_2.5_, along with other inhaled materials, cause the development and exacerbation of respiratory diseases such as asthma, COPD, and severe respiratory infections including SARS-CoV-2 infection [3, 68]. PM_2.5_ is also associated with increased levels of IL-8 and IL-1$\beta$, as well as subsequent increased neutrophil density and total lymphocyte counts in bronchoalveolar lavage fluid (BALF) [19, 39, 77, 85, 86, 90]. Our findings build upon these studies and provide new evidence suggesting that the microvascular endothelium in the ACR is likely an important source of the elevated levels of IL-8 and IL-1$\beta$. Moreover, as IL-8 and IL-1$\beta$ are strong neutrophil attractants, [16, 65, 66], our findings suggest that endothelial IL-8 and IL-1$\beta$ signaling may play an important role in initiating the onset of pulmonary inflammation following a PM_2.5_ exposure.

During the process of immune cell mediated inflammation, NRF2 signaling is typically a cytoprotective, antioxidant and anti-inflammatory response [1, 13, 75]. However, in the absence of immune cells, we observed an increase in early endothelial NRF2 activity (significant protein stability after a 2 h ACRE-DEP exposure) and significant, endothelial NRF2-mediated IL-8 secretion. We propose that early endothelial NRF2 activity and IL-8 secretion serves to recruit neutrophils and other leukocytes to the ACR following an inhalation exposure. Placed at the interface of the respiratory and cardiovascular systems, the capillary endothelium is in an influential position to regulate immune cell activation and recruitment into the respiratory space. As IL-8 is a potent chemokine, modest increases of IL-8 secretion into the capillaries, similar to which we observed, may induce significant inflammatory cell recruitment. Early capillary endothelial NRF2-mediated IL-8 secretion following an ACRE-DEP exposure provides a potential, underexplored mechanism of the initiation of lung inflammation frequently observed in vivo following exposure to inhaled toxicants.

In the absence of a disrupted alveolar epithelial barrier or perceivable DEP translocation across the epithelial barrier (Fig. 1B–D), the observed microvascular endothelial exposure responses are likely driven by secreted alveolar epithelial mediators and metabolites. Our results, illustrating that endothelial NRF2 activation is modulated by epithelial MAPK signaling, supports that secreted alveolar epithelial mediators modulate the endothelial response. Indeed, other studies investigating alveolar epithelial and microvascular endothelial cells have also observed endothelial responses following epithelial cell exposure, in the absence of a disrupted epithelial barrier [8, 9, 40, 44]. While these studies did not investigate the intercellular signaling mechanisms driving the endothelial response, the authors hypothesize that endothelial responses may be the result of secreted alveolar epithelial mediators [8, 9, 40, 44]. MAPK signaling commonly results in the secretion of pro-inflammatory cytokines that can serve as a positive feedback loop to perpetuate MAPK signaling through inter- and intracellular mechanisms [21, 34]. However, we did not find a significant increase in epithelial secretion of the common pro-inflammatory cytokines of MAPK signaling, IL-8, IL-6, IL-1$\beta$, and TNF$\alpha$ [12, 34, 72]. We also did not identify activated MAPK signaling in the endothelium. In addition to MAPK-dependent signaling products directly interacting with the endothelium, epithelial MAPK signaling may also modulate the endothelium through indirect mechanisms. Intracellular MAPK signaling can modulate intracellular NRF2 activity, suggesting secreted intercellular mediators could be a result of epithelial MAPK-induced epithelial NRF2 activity [49, 52, 87]. Indeed, ERK1/2, p38, and NRF2 activity overlapped in the epithelial cells following an ACRE-DEP exposure. However, inhibition of epithelial MAPK activity did not induce a significant decrease in NRF2-dependent transcriptional activity in the epithelial cells (Additional file 3: Figure S6A). Thus, suggesting the endothelial response is not a result of secreted mediators from MAPK-induced epithelial NRF2 signaling. As MAPK signaling is an upstream activator of several cellular responses, proteomic investigation of the basolateral medium collected from the MAPK inhibitor experiment may shed light on the MAPK-mediated intercellular signaling molecule(s).

It is also worthwhile to explore other options of intercellular signaling molecules, particularly as endothelial NRF2 activity was not completely depleted following epithelial MAPK inhibition. Chemical analysis of the DEP used within this study revealed a large organic carbon component, specifically with a large abundance of polyaromatic hydrocarbons (PAH) [81]. In concurrence, “xenobiotic metabolism signaling” and “xenobiotic metabolism AHR signaling pathway”, which included upregulation of *CYP1A1* and *CYP1B1* genes, were among the top activated pathways identified in the endothelial cells following a 24 h ACRE-DEP exposure. Thus, it cannot be ruled out that desorbed or solubilized PAHs could diffuse into the basolateral space after 24 h and activate an endothelial transcriptional response. The innate immune cell receptors, toll-like receptors (TLR), also activate endothelial pro-inflammatory signaling and cytokine production [45]. TLR signaling also induces NRF2 activation and NRF2-related gene expression [17, 31, 58, 61]. Gong et al. observed that microvascular endothelial exposure to the oxidized phospholipid, oxidized 1-palmitoyl-2-arachidonyl-sn-glycero-3-phosphorylcholine (ox-PAPC), a non-infectious TLR agonist, induced the expression of *HMOX1* and *IL-8* [31]. Moreover, Gong et al. observed that the *HMOX1* and *IL-8* expression was comparable to the expression after direct exposure of endothelial cells to DEP. PM exposure is associated with increased oxidized phospholipids [19, 43]. Thus, a TLR agonist, such as oxidized phospholipids, secreted from the alveolar compartment could be an important intercellular mediator driving endothelial NRF2-dependent pro-inflammatory cytokine production. Together, these findings further highlight how cells beyond the epithelial layer can be affected by an epithelial exposure and encourage continued investigation to clarify the intercellular mechanisms driving these effects.

## Conclusions

Our results demonstrate that PM deposition in the ACR elicits a larger transcriptional response in the microvascular endothelium than the directly exposed, alveolar epithelium. Our results reveal that this robust endothelial response is enriched in NRF2 oxidative stress signaling and ROS accumulation that leads to the production and secretion of endothelial IL-8. As increased IL-8 production is linked with the development of several respiratory diseases associated with PM exposure, these findings suggest capillary endothelial proinflammatory signaling may be an important mechanism of PM-induced respiratory disease. Furthermore, we determined that alveolar epithelial MAPK signaling mediates the microvascular NRF2-dependent IL-8 production. To the best of our knowledge this is the first study to mechanistically demonstrate intercellular regulation of NRF2 between the alveolar epithelium and microvascular endothelium in the absence of immune cells and immune cell signaling. While the exact epithelial MAPK mediator is still to be identified, these data emphasize that intercellular crosstalk within the ACR likely plays an influential role in mediating alveolar and endothelial exposure responses. These findings illustrate that investigating inhalation exposure effects on cells beyond the directly exposed epithelium and investigating the mechanisms of intercellular communication between these cells, is an area of research that warrants future investigation. Elucidating these mechanisms in response to an inhaled toxicant has important implications for other studies of disease mechanisms, particularly those involving IL-8 secretion, like acute respiratory disease induced by SARS-CoV-2 infection. More broadly, this work illustrates the utility of tri-culture in vitro models in identifying the biological mechanisms driving inhalation exposure responses to a variety of emerging respiratory threats such as climate change driven wildfire emissions, evolving respiratory viruses, and inhaled PFAS compounds.

## Limitations of the study

The ACRE model was designed using cell lines to increase the affordability and accessibility of the model. While primary endothelial cell donors were incorporated to validate antioxidant gene expression, a limitation of our study is that we have not recapitulated our findings using a combination of primary alveolar epithelial, fibroblast, and microvascular endothelial cells. In addition, our use of only one exposure material limits our ability to evaluate if these findings are DEP-specific or a ubiquitous response to inhaled materials depositing in the ACR. Moreover, while the use of a submerged DEP exposure facilitates the comparison of exposure responses between the ACRE model and other in vitro studies, it also has several limitations. First, it is unclear how representative submerged exposures are of the in vivo exposure scenario. Second, while a nominal dose of 20 µg/cm^2^ was used, this dose may not reflect the actual deposition amount on the epithelial layer, even after a 24 h ACRE-DEP exposure. Third, the comparability between submerged particle deposition on the epithelial cell layer in vitro and inhaled particle deposition on the alveolar epithelial layer in vivo is unknown [28]. These findings warrant future studies to investigate how the cells of the ACR are affected following an air liquid interface (ALI) exposure of DEP, or other particulate matter exposure, and how exposure format affects particle deposition. Lastly, we do not investigate the exposure response in the underlying fibroblast layer or include resident ACR immune cells in our model. Moreover, while we did not observe any overt changes in fibroblast morphology after exposure to the HULEC medium, we cannot rule out the possibility that exposure to this medium elicited unique effects in the IMR90s.

Future studies investigating how fibroblast and immune cell signalling can be incorporated into the ACR exposure response are worthwhile.

## Methods

### Reagents and media

Dexamethasone (Dex) (MilliporeSigma, #D4902) was diluted in Advanced-RPMI (A-RPMI; ThermoFisher, #12633020) basal growth media, stored in 4 °C, and used within 1 month. Human epidermal growth factor (Fisher Scientific, #PHG0311) was resuspended in Dulbecco’s phosphate buffered saline (DPBS; ThermoFisher #14190250) to a concentration of 100 µg/mL, distributed into 60 µL aliquots, stored in -20 °C, and used within 1 year. Hydrocortisone (MilliporeSigma, #H0888), was resuspended in 100% ethanol to a concentration of 5 mg/mL, distributed in 125 µL aliquots, stored in − 80 °C, and used within 1.5 years.

### Preparation of diesel exhaust particulate suspensions

Whole diesel exhaust particulates (DEP) were collected by Sagai et al. [74] and characterized as “A-DEP” by Singh et al. [81]. These particulates have been characterized and used for in vitro exposures in previous studies and [18, 27, 74, 81]. Thus, we sought to use them as a model oxidant particulate to investigate and compare exposure effects in the new model described below. DEP were added to 5 mL of resuspension medium (A-RPMI medium with 1% penicillin/streptomycin (P/S; ThermoFisher, #15140122)) at 5 mg/mL prior to suspension by sonication on a Sonic Dismembrator Model 500 sonicator with microprobe tip (Fisher Scientific) for 2 cycles with the following parameters: 1 min per cycle, 0.9 s on, 0.1 s off, 30% output with mixing by inversion after each cycle. The DEP suspension was then diluted to 1 mg/mL in resuspension medium and single-use 550 µL aliquots were flash frozen in liquid nitrogen prior to storage at − 80 °C until use in experiments. Prior to use, aliquots were thawed at room temperature, vortexed for approximately 5 s, and diluted to 100 µg/mL in basal exposure medium resulting in a 20 µg/cm^2^ exposure scenario (described below). 10 µg/cm^2^ is a relevant in vitro dose comparable to in vivo ambient PM_2.5_ concentration [42]. However, these analyses did not consider the inefficient PM removal from the alveolar space and high retention rates of PM deposition in this region that can persist for weeks [59, 63]. To reflect this increased PM burden and to enable comparisons with current literature studies, we chose to use a 20 µg/cm^2^ DEP exposure [18, 27, 73, 83]. Agglomerate size distribution was determined by flow cytometric comparison with size calibration standards (ThermoFisher #F13838). DEP had an approximate average size of 1 µm in diameter and an upper range of 6 µm in diameter (Fig. 1D).

### Cell culture

The following human cell types were selected to investigate the alveolar capillary region: (1) alveolar epithelial cells, (2) fibroblasts, and (3) lung microvascular endothelial cells.

To represent the alveolar epithelium of the ACR, NCI-H441 cells (hereafter referred to as “H441”; human, male, alveolar-like epithelial cells, American Type Culture Collection (ATCC) #CRM-HTB-174, batch #F-14929) were purchased from the University of North Carolina-Chapel Hill Tissue Culture Core Facility and used for exposures within an adjusted population doubling (APD; described in [27]) range of 4–27. While A549 cells are often used to model the alveolar epithelial barrier, they do not form tight junctions, a key characteristic of the alveolar epithelium in vivo [9, 10, 30]. Thus, we selected the H441 cell line because it forms a functional, tight junction-mediated epithelial barrier and maintains properties of both alveolar type 1 and type 2 cells, as well as other characteristics of the alveolar epithelium in vivo [76]. To represent the interstitial fibroblasts of the ACR, the IMR90 cell line (human lung fibroblasts,ATCC #CCL-182, batch #64155514 was selected as it is a human lung fibroblast cell line that grows well with other cell types in tri-culture models [27]. IMR90 cells were used for exposures within an APD range of 3–15. IMR90 and H441 were maintained in A-RPMI growth medium (A-RPMI supplemented with 5% Fetal bovine serum (FBS,ThermoFisher, #16000044, certified), 0.5% P/S and 4 mM GlutaMAX supplement (ThermoFisher #35050061). To represent the lung microvascular endothelial cells, HULEC-5a (hereafter referred to as “HULEC”; human lung microvascular endothelial cells, ATCC #CRL-3244, batch #70025430 and batch #70001024) and primary lung microvascular endothelial cells (PMVEC) (*n* = *3* donors) were selected. The HULEC cell line was selected as it is one of the only human lung microvascular endothelial cell lines available that expand in culture similar to other immortalized cell lines and because it is readily, publicly available. HULEC were used for exposures within an APD range of 3–12 and were maintained in complete HULEC growth medium, MCDB 131 Medium (MCDB), no glutamine (Gibco #10372019) supplemented with 10% FBS, 10 mM GlutaMAX, 1% P/S, 10 ng/mL human epidermal growth factor, and 1 µg/mL Hydrocortisone). PMVEC (*n* = *3* donors) were incorporated to investigate endothelial responses in non-immortalized cells. PMVECs were generously provided by Dr. Scott Randell of the UNC Cystic Fibrosis/Pulmonary Research and Treatment Center Tissue Procurement Core and used within an APD range of 7–12. PMVECs were maintained in LONZA EGM-2-MV growth medium (LONZA, #CC-3162) that was prepared according to the manufacturer’s instructions. Donor demographics are listed in Additional file 2: Table S3. All cell types were grown in a humidified cell culture incubator at 37 ˚C with 5% CO_2_ and ambient O_2_ levels (hereafter referred to as a “tissue culture incubator”). All cells were passaged onto tissue culture plates coated with bovine type I collagen solution (Advanced BioMatrix, San Diego, CA, #5005) as described in the methods document by [56]. All cell culture and experiments were conducted at ambient oxygen levels.

### Alveolar capillary region exposure model (ACRE Model) setup

ACRE Model plating formats for all downstream applications, cell seeding densities, collagen coating volumes, media volumes, and product numbers can be found in Additional file 2: Table S4. On day 1 of the ACRE setup (illustrated in Additional file 3: Figure S7), IMR90s were seeded on the bottom surface of inverted Transwell^®^ inserts, collagen-coated at 50 µg/mL, with a 0.4 µm pore size (selected to prevent cell migration across the Transwell membrane) and incubated at 37 ˚C with 5% CO_2_ and 20% O_2_ for 2 h to permit cell attachment. Excess media and unattached cells were then removed by gentle aspiration and seeded inserts were returned to their hanging position inside the appropriate multi-well plates. H441 cells were then seeded on the collagen-coated apical side of the Transwell^®^ inserts in A-RPMI growth medium and A-RPMI growth medium was added to each well. Co-cultures were then placed in a tissue culture incubator for 24 h. On day 2, the co-culture basolateral medium was replaced with fresh A-RPMI growth medium and the apical medium was replaced with polarization medium (A-RPMI growth medium supplemented with 0.5 µM dexamethasone). On day 3, the polarization medium in the apical compartment was refreshed. On day 4, HULECs were seeded in separate collagen-coated multi-well tissue culture plates in HULEC growth medium and placed in a tissue culture incubator for 9 h to permit attachment and the formation of a confluent monolayer. The medium was then aspirated and replaced with HULEC exposure medium (MCDB-131 supplemented with 1% FBS, 1% P/S, and 10 mM GlutaMAX). The apical and basolateral media from the seeded Transwell^®^ inserts were aspirated from co-culture inserts prior to the gentle transfer to HULEC-seeded wells. Polarization medium was then added to the apical side of the Transwell^®^ inserts prior to the tri-cultures being placed in a tissue culture incubator for 14 h. On day 5, the basolateral medium of the tri-culture was replaced with fresh HULEC exposure medium. The apical medium was also replaced with either basal exposure medium (A-RPMI supplemented with 0.5% P/S, 0.5 µM dexamethasone, and 4 mM GlutaMAX) (vehicle, VEH) or exposure medium containing diesel exhaust particulate (DEP) suspension. DEP suspensions of 100 µg/mL, which corresponds to 20 µg/cm^2^ (74 µL for 6.5 mm inserts, 250 µL for 12 mm inserts and 1000 µL for 24 mm inserts), were used for exposures. The tri-culture was then incubated at 37˚C with 5% CO_2_ for the indicated exposure duration. Following incubation, apical and basolateral compartments were then separated for cell-specific analysis. Basolateral medium was also collected into 1 mL aliquots, centrifuged at 13,000 × *g* to pellet large cell debris, and the supernatant was transferred to new collection tubes.

### Whole-mount immunofluorescent (IF) staining

Cells were seeded in 12 mm Transwell^®^ inserts as indicated in the ACRE Model Setup and fixed for IF staining after 24 h exposure to VEH or ACRE-DEP. Prior to staining, ACRE inserts were placed in new multi-well plates and apical and basolateral sides of the inserts were rinsed in DPBS. Both sides of the ACRE inserts were fixed in 4% paraformaldehyde (Electron Microscopy Sciences Part #15710) for 20 min at room temperature, rinsed in DPBS, and blocked with blocking buffer (4% Bovine serum albumin (BSA) (Millipore Sigma, #A7906), 1% Fish Gelatin (Gelatin from cold water fish skin (Millipore Sigma, #G7765), and 0.3% Triton X-100 (Millipore Sigma, #X100) in DPBS, hereafter referred to as 100% blocking buffer) for 1 h. Both sides of the ACRE inserts were then incubated with α-ZO-1 or α-E-Cad overnight at 4 ˚C in 100% blocking buffer. Cells were washed with 25% blocking buffer in DPBS and both sides of the ACRE inserts were incubated with Alexa Fluor 488-conjugated donkey α-rabbit IgG (H + L) (ThermoFisher, #A21206) in 100% blocking buffer for 2 h at room temperature, protected from light. Cells were then counterstained with a solution of 50 µg/mL Hoechst 33342 (ThermoFisher, #H3570, 1/200 dilution) and 0.07 µM Alexa Fluor 594 Phalloidin (ThermoFisher, #A12381, 1/1000 dilution) in DPBS for 30 min at room temperature, protected from light. The Transwell^®^ membrane was carefully cut out of the insert and mounted with Molecular Probes ProLong Gold Antifade Mountant (ThermoFisher #P36930) on a glass microscope slide (Fisher Scientific #12-544-1) with No.1.5 coverslips (ThermoFisher #3405) and the coverslip edges were sealed with nail polish. Slides with mounted coverslips were temporarily stored at room temperature protected from light for 24 h to permit drying prior to long term storage at 4 ˚C. Slides were imaged on a Nikon Eclipse Ti C1/C2 Confocal Microscope, using the 20 × objective and the Nikon software Nis-Elements. Three randomly selected fields of view for each condition were imaged. Scale bars represent 100 µm. Excitation and emission wavelengths were 408/450 nm for Hoescht 33342, 488/515 nm for AlexaFluor 488 and 561/605 nm for AlexaFluor 594. Antibody dilutions and product information are listed in Additional file 2: Table S5.

### Trans-epithelial electrical resistance (TEER) assay

Cells were seeded in 12 mm Transwell^®^ inserts as indicated in the ACRE Model Setup. Following the indicated exposure duration, ACRE inserts were transferred to new multi-well plates and rinsed in pre-warmed (37 °C) DPBS. DPBS was removed and ACRE inserts were transferred to the EndOhm-12 Chamber Cup (World Precision Instruments Inc, Sarasota, FL, USA) containing 3 mL of pre-warmed (37 °C) A-RPMI growth medium. 500 µL of pre-warmed (37 °C) A-RPMI growth medium was added to the apical compartment and TEER measurements were taken according to [82]. Unseeded, non-collagen coated, Transwell^®^ inserts were used to determine background resistance. TEER values were calculated by subtracting the background resistance from the ACRE unseeded, non-collagen coated insert and then multiplying by the insert surface area (in cm^2^). TEER was determined for 24 h VEH and 24 h ACRE-DEP conditions. Values represent the mean (± SD) from three independent experiments, each of which was performed in technical triplicate (*i.e.*, three inserts per independent experiment for each condition). Statistical analysis was conducted in GraphPad Prism (version 9.3.1) using a two-tailed, unpaired t-test.

### Endothelial cell viability

Cells were seeded in 12 mm Transwell^®^ inserts as indicated in the ACRE Model Setup. Prior to viability analysis, ACRE inserts were transferred to new multi-well plates. All cells were rinsed with DPBS. A 2X-stock solution of the LIVE/DEAD reagents, 0.75 µl/mL of calcein AM (4 mM) and 1.5 µL/mL ethidium homodimer-1 (2 mM) (ThermoFisher #L3224), was prepared in HULEC Live Cell Imaging Medium (MCDB-131 with 4 mM GlutaMAX) and in ACRE Insert Live Cell Imaging Medium (Basal A-RPMI). Both solutions were then diluted two-fold in either HULEC Live Cell Imaging Medium or ACRE Insert Live Cell Imaging Medium, respectively. Hoechst 33,342 was then added (1/200 dilution) to each medium at a final concentration of 50 µg/mL. The diluted HULEC Live Cell Imaging solution was added to the seeded HULEC and the diluted ACRE Insert Live Cell Imaging solution was added to the apical and basolateral compartments of the ACRE inserts. The seeded cells were incubated with LIVE/DEAD reagents for 40 min at room temperature, protected from light. Cells were then analyzed at excitation/emission 375/460 nm (Hoescht), 480/535 nm (LIVE), and 560/635 nm (DEAD) on a ImageXpres Micro 4 (IXM4) High-Content Imaging System (Molecular Devices). Transwell^®^ inserts and HULEC were tested after 24 h VEH and 24 h ACRE-DEP exposures. Extra HULEC and ACRE inserts were seeded for dead cell controls to obtain maximum DEAD signal. Positive DEAD controls were prepared according to the manufacturer’s protocol by incubation in 70% ethanol for 30 min at room temperature before following the aforementioned LIVE/DEAD staining procedure. Quantification of dead cells was determined through the LIVE/DEAD run analysis within the IXM4 software. Values represent the average % dead cells (± SD) from three independent experiments, which were performed in technical triplicate (*i.e.*, three inserts per independent experiment for each condition), and normalized to maximum DEAD signal. All normalized viability measurements were statistically compared to the appropriate vehicle with an ordinary one-way ANOVA and Šidák’s multiple comparisons post-hoc test in GraphPad Prism (version 9.3.1).

### ACRE Model Adenylate Kinase (AK) Cytotoxicity Assay

Cells were seeded in 12 mm Transwell^®^ inserts as indicated in the ACRE Model Setup. Following a 24 h VEH or ACRE-DEP exposure, 1 mL of conditioned basolateral medium was collected, aliquoted, and stored at -80 ˚C until ready for analysis. Upon analysis, frozen VEH and ACRE-DEP basolateral medium samples were thawed and AK reagents were brought to room temperature. The AK detection reagent and assay buffer (AKDR) were prepared as instructed by the manufacturer. 20 µL of thawed basolateral media samples were mixed with 100 µL of prepared AKDR in a 96-well plate and incubated for 5-min at room temperature. Luminescence of the samples were read using a ClarioStar Plus with an integrated read time of 1 s. Luminescence of the basolateral medium collected from H441 cells seeded in a Transwell^®^ insert and treated with 1% TX-100 for 30 min served as a positive dead control. Average raw luminescence per experimental condition was calculated across three technical replicates. Average raw luminescence was then normalized to the positive dead control average luminescence. The data shown represent the mean (± SD) of three independent experiments. Statistical analysis was conducted in GraphPad Prism (version 9.3.1) using a two-tailed, unpaired t-test.

### RNA-sequencing

Cells were seeded in 12 mm Transwell^®^ inserts as indicated in the ACRE Model Setup. After 6 h and 24 h VEH and ACRE-DEP exposure, cells were rinsed in DPBS. Before collection of H441 total RNA, a Kimwipe was used to gently rub off and remove the IMR90 fibroblasts from the underside of the ACRE insert. Total RNA was then extracted from the and HULEC using the Zymo Quick-RNA Miniprep Kit (Zymo Research, #R1055) and quantified using a Nanodrop OneC nanospectrophotometer (ThermoFisher). Total RNA concentration and quality was validated by running an RNA broad range Qubit (Thermo Fisher Scientific, #Q10211) and from an Agilent Tapestation 4200 (Agilent, Model #G2991BA), respectively, at the UNC High Throughput Sequencing core. cDNA libraries for the H441 and HULEC RNA-seq analysis were generated at the UNC High Throughput Sequencing core using the KAPA Stranded mRNA-Seq Kit, with KAPA mRNA Capture Beads (Roche Sequencing and Life Science, #07962207001) to enrich for polyadenylated species. Libraries underwent quality control via dsDNA High Sensitivity Qubit (Thermo Fisher Scientific, #Q32851) and Agilent Tapestation 4200. Libraries were processed on a MiSeq Nano flowcell (MS-103-1001) and paired-end sequencing with 2 × 150 cycles was performed on the Illumina Novaseq 6000 platform with an average sequencing depth of 42.5 million and 41.25 million paired end reads per H441 and HULEC library, respectively. Mapping of sequence reads to the human genome (GRCh38) was performed by STAR (version 2.5) with the following parameters: -outSAMattrIHstart 0-outFilterType BySJout-alignSJoverhangMin 8-outMultimapperOrder Random (other parameters at default settings). Mapped read counts per gene were collected by Subread feature Counts (version 1.5.0-0-p1). Genes with a minimum average of normalized mapped read counts > 50 in at least one category were selected for differential gene expression analysis. Differential gene expression analysis was conducted using DESeq2 (v1.34.0) with filtering thresholds of FDR < 0.01 and |fold change|> 2 to normalize gene expression and identify differentially expressed genes. DESeq2 was also used to generate a PCA plot for visualizing sample similarity and calculating sample-to-sample distances for normalized counts from variance stabilizing transformation. Ingenuity Pathway Analysis (IPA) was used to identify the top canonical pathways in the H441 and HULEC following a 6 h and 24 h VEH and ACRE-DEP exposure using the differentially expressed genes that had at least an adjusted p-value of 0.05 and a 0.6 log2fold change. The top 6 canonical pathways with a -log(p-value) of at least 2 and with at least 4 differentially expressed genes contributing to their calling were selected.

### qPCR validation

Cells were seeded in 12 mm Transwell^®^ inserts as indicated in the ACRE Model Setup. After completion of desired VEH and ACRE-DEP exposure duration, inserts were apically rinsed in DPBS. Before collection of H441 total RNA, a Kimwipe was used to gently wipe the IMR90 fibroblasts from the underside of the ACRE insert. Total RNA was then extracted from the H441 and HULEC using the Zymo Quick-RNA Miniprep Kit (Zymo Research, #R1055) and quantified using a Nanodrop OneC nanospectrophotometer (ThermoFisher). Complimentary DNA (cDNA) was synthesized from 500 ng of total RNA using the iScript reverse transcription kit (Bio-Rad, #1708841) according to the manufacturer’s protocol. Transcript abundance was assessed by quantitative PCR using multiplexed primers, hydrolysis probes (sequences listed in Additional file 2: Table S6) and iTaq Universal Probes Supermix (BioRad, #1725135) in technical triplicates on a CFX96 Touch (Bio-Rad). Fold change values between VEH and ACRE-DEP treatments were calculated and normalized to sample-matched changes in the reference gene β-actin (ACTB) using the Pfaffl method [67]. The data shown represent the mean (± SD) of three independent experiments. For each experiment, lysates from three inserts were pooled for each condition prior to RNA extraction. The resulting samples from each experiment were then assayed in technical triplicate.

### Western blotting

Cells were seeded in 12 mm Transwell^®^ inserts as indicated in the ACRE Model Setup. After completion of desired VEH and ACRE-DEP exposure duration, inserts were apically rinsed in DPBS. Before collection of H441 protein, a Kimwipe was used to gently remove IMR90 fibroblasts from the underside of the ACRE insert. H441 cell lysate was then collected in RIPA Buffer (50 mM Tris, pH 8.0; 150 mM NaCl; 1% Triton X-100; 400 μM EDTA; 10% glycerol; 0.1% SDS; 0.1% deoxycholate) with 1X cOmplete protease inhibitor cocktail (Millipore Sigma, #4693116001) and 1X PhosSTOP phosphatase inhibitor (Millipore Sigma, #4906845001), transferred into 1.5 mL Eppendorf tubes and incubated on ice for 20 min to generate cellular protein extracts. Cellular protein extracts were then clarified by centrifugation at 14,000 × *g* for 15 min at 4 °C. Supernatants were transferred to new Eppendorf tubes on ice and three 10 µL aliquots were removed and stored in individual Eppendorf tubes at − 80 °C until use for total protein quantification by BCA assay (ThermoFisher, #23225). The remaining supernatant was supplemented with 5X Laemmli loading buffer (250 mM Tris, pH 6.8; 500 mM DTT; 50% glycerol; 10% SDS; 0.5% bromophenol blue) to a final concentration of 1X, incubated at 95 °C for five min, and aliquoted prior to storage at − 80 °C until use. For each sample, equal amounts of total protein were loaded into 10% SDS-PAGE gels, electrophoresed, and electrotransferred (100v for 60 min) to 0.45 µm nitrocellulose membranes (Bio-Rad, #1620115) via wet transfer (Grant Transfer Buffer: 48 mM Tris-Base, 39 mM Glycine, 20% Methanol). Membranes were blocked at room temperature for 1 h using 5% BSA in 1X TBST and were incubated in a 1:1000 primary antibody solution in blocking buffer overnight at 4 °C while rocking. Following primary antibody binding, membranes were washed three times for five min in 1X TBST and then incubated in a 1:10,000 secondary antibody, peroxidase AffiniPure F(ab')_2_ Fragment Donkey Anti-Rabbit IgG (H + L) (Jackson ImmunoResearch, #711-036-152), solution in blocking buffer for 1 h at room temperature. Membranes were washed three times for five min in 1X TBST and imaged on a ChemiDoc MP Imaging System (BioRad) using Clarity Max Western ECL Substrate (BioRad, #1705062) to generate chemiluminescent images. Densitometry was performed using BioRad Image Lab version 6.1 software. Densitometry values represent the mean from three independent experiments ± SD. Statistical analysis was conducted in GraphPad Prism (version 9.3.1) using multiple unpaired t-tests and Šidák’s multiple comparisons post-hoc test. Antibodies can be found in Additional file 2: Table S5 and additional, specific western blotting details can be found in [27].

### HULEC siRNA reverse transfection

Cells were seeded in 12 mm Transwell^®^ inserts as indicated in the ACRE Model Setup. Before plating the HULEC on day 3 of the ACRE Model Setup, ON-TARGETplus h-NRF2 (Horizon Discovery, #L003755-00-0005) and Non-targeting Control Pool (Horizon Discovery, #D-001810–10-20) were diluted in Opti-MEM | Reduced Serum Medium (Gibco, #31985070) and Lipofectamine® 2000 to permit siRNA complex formation in 12-well multi-well plates. After complex formation, HULEC were added in HULEC growth medium, resulting in a final siRNA concentration of 50 nM. HULEC were placed in a tissue culture incubator for 9 h to permit attachment and the formation of a confluent monolayer. Following incubation, the medium was aspirated and ACRE inserts were combined with seeded HULEC and exposed as described in the ACRE Model Setup. qPCR and western blot analysis was performed on HULEC cellular RNA and protein extracts after 2 h VEH and 2 h ACRE-DEP treatment for investigation of knockdown efficiency. qPCR analysis was performed on synthesized HULEC cDNA after 6 h VEH and 6 h ACRE-DEP treatment for investigation of NRF2 knockdown effects on downstream NRF2 targets. Cell seeding densities, media volumes, and product numbers can be found in Additional file 2: Table S4 and siRNA oligonucleotide sequences can be found in Additional file 2: Table S6. Statistical analysis was conducted in GraphPad Prism (version 9.3.1) using an ordinary one-way ANOVA and Šidák’s multiple comparisons post-hoc test.

### Alveolar MAPK chemical inhibition pre-treatment

Cells were seeded in 12 mm Transwell^®^ inserts as indicated in the ACRE Model Setup. On day 4 of the ACRE Model Setup, prior to exposure, seeded ACRE inserts were transferred to a temporary multi-well plate. Pre-warmed (37 ˚C) basal exposure medium containing the ERK1/2 and p38 MAPK inhibitors, 800 nM SCH772984 (Cayman Chemical, #19166) and 300 nM LY2228820 (mesylate) (Cayman Chemical, #23259), respectively, was added to the apical and basolateral compartments of the multi-well plate. ACRE inserts were returned to the tissue culture incubator for 2.5 h. Following incubation, basal exposure medium containing MAPK inhibitors was removed, and ACRE inserts were combined with seeded HULEC and exposed as described in ACRE Model Setup. Western blot analysis was performed on H441 cellular protein extracts after 1 h VEH and 1 h ACRE-DEP treatment and qPCR analysis was performed on synthesized HULEC cDNA after 6 h VEH and 6 h ACRE-DEP treatment. This methodology was then repeated with an additional set of ERK1/2 and p38 inhibitors, 1 µM BVD523 and 3 µM SB203580, respectively. Statistical analysis was conducted in GraphPad Prism (version 9.3.1) using an ordinary one-way ANOVA and Šidák’s multiple comparisons post-hoc test.

### Meso scale discovery (MSD)—enzyme linked immunosorbent assay (ELISA)

Cells were seeded in 12 mm Transwell^®^ inserts as indicated in the ACRE Model Setup. After exposure of 6 h VEH and 6 h ACRE-DEP exposure, basolateral medium was collected from each well and centrifuged at 4 °C for 10 min at 13,000 × *g* to remove cell debris. The supernatant was transferred to new collection tubes and stored in 1 mL aliquots at − 80 °C. Levels of IL-8 in the basolateral medium was quantified using the Meso Scale Diagnostics V-PLEX Pro-inflammatory Panel II (MSD, #K15053D-1; IL-8 dynamic range of detection of 0.07–375 pg/mL) according to the manufacturer’s instructions. The data shown represent the mean (± SD) calculated concentration of IL-8 from three independent experiments, with each independent experiment assayed in technical triplicate per experimental condition. Statistical analysis was conducted in GraphPad Prism (version 9.3.1) using an ordinary one-way ANOVA and Šidák’s multiple comparisons post-hoc test.

### Supplementary Information

**Additional file 1**.** Table S1**. Top predicted canonical pathways enriched amongst the H441 and HULEC following a 6 and 24 h ACRE-DEP exposure.

**Additional file 2. Table S2**. NRF2 knock down in endothelial cells using siRNA molecules targeting NRF2. mRNA expression of NRF2 in endothelial cells following a 6 h ACRE-DEP exposure. Values represent mean fold change normalized to Β-Actin, relative to VEH. n=3 independent experiments ± SD. ****p ≤ .0001.** Table S3**. Human primary lung microvascular endothelial cell (PMVEC) donor demographics and cause of death. All PMVEC donors were healthy, non-smokers. **Table S4**. ACRE Model Downstream Application Setup. All ACRE Model experiments were plated in the 12 mm Transwell Insert format with the exception of the downstream applications bolded below. The number of experimental replicates conducted for each application is specified in brackets. Endothelial plating densities applies to both HULEC and pMVEC seeding. * Indicates basolateral medium volume and seeding density parameters optimized for HULEC siRNA Reverse Transfection. Abbreviations: Basolateral compartment, B.C. **Table S5.** Antibody product numbers and dilutions used.** Table S6**. Oligonucleotide sequences of primers and probes used in qPCR and of the siRNA used for reverse transfection.

**Additional file 3. Figure S1.** ACRE Model Viability. (A) Total viability of the ACRE model exposed to VEH or DEP for 24 H. All viability measures were normalized to the dead control and statistically compared to the appropriate vehicle. Values represent the mean of n=3 independent experiments ± SD. *p ≤ .05.** Figure S2**. Epithelial and Endothelial p65 Expression. (A) Protein expression and densitometry of the phospho-p65 (p-p65) and total p65 (p65) in the epithelial cells over a 2 – 24 h ACRE-DEP exposure. (B) Protein expression and densitometry of the phospho-p65 (p-p65) and total p65 (p65) in the endothelial cells over a 2 – 24 h ACRE-DEP exposure. (A-B) Values represent the mean of n=3 independent experiments ± SD and immunoblots are representative images from n=3 independent experiments.** Figure S3**. ROS Accumulation. (A) ROS accumulation in endothelial cells over a 24 h ACRE-DEP exposure. Values represent the mean of n=3 independent experiments ± SD. *p ≤ .05.** Figure S4**. Epithelial cell MAPK inhibition pre-treatment with the ERK1/2 and p38 inhibitors, BVD-523 and SB203580, respectively. (A) mRNA expression of the antioxidants HMOX1, NQO1, and GCLM in endothelial cells after a 6 h ACRE-DEP exposure. (B) mRNA expression of IL-8 in endothelial cells after a 6 h ACRE-DEP exposure. (A-B) Values represent the mean of n=3 independent experiments ± SD. Statistically significant differences between VEH, ACRE-DEP, and DEP + ERKi/p38i cells are indicated by **p ≤ .01, ***p ≤ .001, and ****p ≤ .0001.** Figure S5**. (A) IL-1β, TNF-α and IL-6 secretion in the basolateral medium of the ACRE and ARE model after a 6 h ACRE-DEP exposure. Values represent the mean of n=3 independent experiments ± SD. ****p ≤ .0001.** Figure S6**. (A) mRNA expression of HMOX1, NQO1, GCLM and IL-8 expression in the epithelial cells after a 6 h ACRE-DEP exposure and epithelial cell MAPK inhibition pre-treatment. Values represent the mean of n=3 independent experiments ± SD. Statistically significant differences between VEH, ACRE-DEP, and DEP + ERKi/p38i cells are indicated by *p ≤ .05 and **p ≤ .01.** Figure S7**. ACRE Model Setup. (A) ACRE model setup using Transwell® inserts.

**Additional file 4. Figure S8**. Full length blots of all western blot images used in the manuscript.

**Additional file 5. Method S1**. CellROX Green HULEC Reactive Oxygen Species Detection.

## Data Availability

The RNA-sequencing datasets supporting the conclusions of this article are available in the NCBI Gene Expression Omnibus (GEO) repository, #GSE229544 and https://www.ncbi.nlm.nih.gov/geo/query/acc.cgi?acc=GSE229544. The remaining datasets used in this study are publicly available through EPA’s ScienceHub.
