# Supplementary material for: Epithelial MAPK signaling directs endothelial NRF2 signaling and IL-8 secretion in a tri-culture model of the alveolar-microvascular interface following diesel exhaust particulate (DEP) exposure
Source: Part Fibre Toxicol. 2024 Mar 11;21:15. doi: 10.1186/s12989-024-00576-8 (PMC10926573; doi:10.1186/s12989-024-00576-8)
Supplement: Supplementary file 2 — Additional file 2. Table S2. NRF2 knock down in endothelial cells using siRNA molecules targeting NRF2. mRNA expression of NRF2 in endothelial cells following a 6 h ACRE-DEP exposure. Values represent mean fold change normalized to Β-Actin, relative to VEH. n=3 independent experiments ± SD. ****p ≤ .0001. Table S3. Human primary lung microvascular endothelial cell (PMVEC) donor demographics and cause of death. All PMVEC donors were healthy, non-smokers. Table S4. ACRE Model Downstream Application Setup. All ACRE Model experiments were plated in the 12 mm Transwell Insert format with the exception of the downstream applications bolded below. The number of experimental replicates conducted for each application is specified in brackets. Endothelial plating densities applies to both HULEC and pMVEC seeding. * Indicates basolateral medium volume and seeding density parameters optimized for HULEC siRNA Reverse Transfection. Abbreviations: Basolateral compartment, B.C. Table S5. Antibody product numbers and dilutions used. Table S6. Oligonucleotide sequences of primers and probes used in qPCR and of the siRNA used for reverse transfection. [file 12989_2024_576_MOESM2_ESM.docx]

**Supplementary Tables**

Table S2

|  | Mean Fold Change | SD | Significance Relative to DEP |
| --- | --- | --- | --- |
| VEH | 1.00 | 0.00 | ns |
| DEP | 1.07 | 0.16 | ns |
| DEP + siNRF2 | 0.09 | 0.02 | **** |
| DEP + SCR | 1.07 | 0.25 | ns |

Table S3

| Donor Catalog # | Sex | Donor Age | Ethnicity | Cause of Death |
| --- | --- | --- | --- | --- |
| MV068kP5 | Female | 56 | Caucasian | Anoxia, 2^nd^ cardiovascular stroke |
| MV079Kp5 | Male | 52 | Caucasian | Head trauma, 2^nd^ gunshot wound |
| MV036Kp5 | Male | 58 | Caucasian | Intracranial Hemorrhage |

Table S4

| **ACRE Model Downstream Application Setup** | | |  |
| --- | --- | --- | --- |
|  | **Western Blotting [3]** | Other Experiments [3] | **CellROX Green [3]** |
| *Transwell Insert* | *24 mm insert (Corning #3450)* | *12 mm insert (Corning #3460)* | *6.5 mm insert (Corning #3470)* |
| *Multiwell Plate* | 6-well Plate (Corning #3506) | 12-well Plate (Corning #3512) | 24-well Plate (Corning #3524) |
| *Insert (Apical Compartment) Growth Area* | 4.67 cm^2^ | 1.12 cm^2^ | 0.33 cm^2^ |
| *Well (B.C.) Growth Area* | 9.5 cm^2^ | 3.8 cm^2^ | 1.9 cm^2^ |
| *Apical Growth Media Volume* | 2000 µL | 500 µL | 100 µL |
| *Apical Polarization Media Volume* | 2000 µL | 500 µL | 100 µL |
| *Apical DEP Volume* | 1000 µL | 250 µL | 74 µL |
| *Basolateral Growth Media Volume* | 2000 µL | 1000 µL  500 µL * | 500 µL |
| *Basolateral Exposure Media Volume* | 2000 µL | 1000 µL  500 µL * | 500 µL |
| *Collagen Coating Volume* | 1000 µL | 250 µL | 74 µL |
| *Inverted Plating Volume* | 1000 µL | 250 µL | 74 µL |
| *Diluted IMR90 Cell Suspension* | 2.9 x 10^4^ cells / mL | 2.8 x 10^4^ cells / mL | 2.78 x 10^4^ cells / mL |
| *IMR90 Cell Density* | 6.25 x 10^3^ / cm^2^ | 6.25 x 10^3^ / cm^2^ | 6.25 x 10^3^ / cm^2^ |
| *IMR90 Cell Count* | 29,000 cells / insert | 7,000 cells / insert | 2063 cells / insert |
| *Diluted H441 Cell Suspension* | 1.75 X 10^5^ cells / mL | 16.8 X 10^4^ cells / mL | 24.8 X 10^4^ cells / mL |
| *H441 Cell Density* | 7.5 X 10^4^ cells / cm^2^ | 7.5 X 10^4^ cells / cm^2^ | 7.5 X 10^4^ cells / cm^2^ |
| *H441 Cell Count* | 3.5 X 10^5^ cells / insert | 8.4 X 10^4^ cells / insert | 2.48 X 10^4^ cells / insert |
| *Diluted Endothelial Cell Suspension* | 1.38 x 10^5^ cells / mL | 1.12 x 10^5^ cells / mL  4 x 10^5^ cells / mL* | 1.1 x 10^5^ cells / mL |
| *Endothelial Cell Density* | 2.9 x 10^4^ cells / cm^2^ | 2.9 x 10^4^ cells / cm^2^  5.18 x 10^4^ cells / cm^2^ * | 2.9 x 10^4^ cells / cm^2^ |
| *Endothelial Cell Count* | 275,500 cells / B.C. | 112,000 cells / B.C.  200,000 cell / B.C.* | 55,100 cells / B.C. |

Table S5

| **Target** | **Manufacturer** | **Item Number** | **Dilution Used** |
| --- | --- | --- | --- |
| α-Alexa Fluor 488 donkey a-rabbit IgG secondary antibody | ThermoFisher Scientific | A21206 | 1:1000 |
| α-ZO1 | Cell Signaling Technology | 13663S | 1:400 |
| α-E-Cadherin | Cell Signaling Technology | 3195S | 1:400 |
| Peroxidase AffiniPure F(ab')₂ Fragment Donkey Anti-Rabbit IgG (H+L) | Jackson ImmunoResearch | 711-036-152 | 1:10,000 |
| α-HMOX1 | Cell Signaling Technology | 82206 | 1:1,000 |
| α-NQO1 | Cell Signaling Technology | 62262 | 1:1,000 |
| α-GCLM | Abcam | ab126704 | 1:1,000 |
| α-GCLC | Abcam | ab190685 | 1:1,000 |
| α-GAPDH | Cell Signaling Technology | 5174 | 1:1,000 |
| α-NRF2 | Cell Signaling Technology | 12721S | 1:1,000 |
| α-Phospho ERK | Cell Signaling Technology | 4370S | 1:1,000 |
| α-Total ERK | Cell Signaling Technology | 4695S | 1:1,000 |
| α-Phospho P38 | Cell Signaling Technology | 4511S | 1:1,000 |
| α-Total P38 | Cell Signaling Technology | 9212S | 1:1,000 |
| α-Phospho P65 | Cell Signaling Technology | 3033S | 1:1,000 |
| α-Total P65 | Cell Signaling Technology | 8242S | 1:1,000 |

Table S6

| **Gene ID** | **Primer/Probe Sequence (5’ – 3’)** | **Manufacturer** |
| --- | --- | --- |
| ACTB | Forward: CTGGCACCCAGCACAATG  Reverse: GCCGATCCACACGGAGTACT  Probe: ATCAAGATCATTGCTCCTCCTGAGCGC | Integrated DNA Technologies |
| HMOX1 | Forward: GAGGGTGATAGAAGAGGCCAAGA  Reverse: GGTCAGCAGCTCCTGCAACT  Probe: TGCGTTCCTGCTCAACATCCAGCTC | Integrated DNA Technologies |
| NQO1 | Forward: TGCAGCGGCTTTGAAGAAG  Reverse: CTTCAGTTTACCTGTGATGTCCTTTC  Probe: GTCCGACTCCACCACCTCCCATCCT | Integrated DNA Technologies |
| GCLM | Forward: CAGACGGGGAACCTGCTG  Reverse: CATCTGGAAACTCCCTGACCA  Probe: GAAGTGCCCGTCCACGCACA | Integrated DNA Technologies |
| GCLC | Forward: CACCCTCGCTTCAGTACCTT  Reverse: CCGGCTTAGAAGCCCTTGAA  Probe: CCGACATAGGAGAGGAGAAAAGGTTGT | Integrated DNA Technologies |
| IL8 | Forward: TTGGCAGCCTTCCTGATTTC  Reverse: TATGCACTGACATCTAAGTTCTTTAGC  Probe: CCTTGGCAAAACTGCACCTTCACACA | Integrated DNA Technologies |
| ON-TARGETplus Non-targeting Pool | 1) UGGUUUACAUGUCGACUAA  2) UGGUUUACAUGUUGUGUGA  3) UGGUUUACAUGUUUUCUGA  4) UGGUUUACAUGUUUUCCUA | Dharmacon |
| ON-TARGETplus NRF2 Smart Pool | 1) UAAAGUGGCUGCUCAGAAU  2) GAGUUACAGUGUCUUAAUA  3) UGGAGUAAGUCGAGAAGUA  4) CACCUUAUAUCUCGAAGUU | Dharmacon |
