# Supplementary material for: Epithelial MAPK signaling directs endothelial NRF2 signaling and IL-8 secretion in a tri-culture model of the alveolar-microvascular interface following diesel exhaust particulate (DEP) exposure
Source: Part Fibre Toxicol. 2024 Mar 11;21:15. doi: 10.1186/s12989-024-00576-8 (PMC10926573; doi:10.1186/s12989-024-00576-8)
Supplement: Supplementary file 3 — Additional file 3. Figure S1. ACRE Model Viability. (A) Total viability of the ACRE model exposed to VEH or DEP for 24 H. All viability measures were normalized to the dead control and statistically compared to the appropriate vehicle. Values represent the mean of n=3 independent experiments ± SD. *p ≤ .05. Figure S2. Epithelial and Endothelial p65 Expression. (A) Protein expression and densitometry of the phospho-p65 (p-p65) and total p65 (p65) in the epithelial cells over a 2 – 24 h ACRE-DEP exposure. (B) Protein expression and densitometry of the phospho-p65 (p-p65) and total p65 (p65) in the endothelial cells over a 2 – 24 h ACRE-DEP exposure. (A-B) Values represent the mean of n=3 independent experiments ± SD and immunoblots are representative images from n=3 independent experiments. Figure S3. ROS Accumulation. (A) ROS accumulation in endothelial cells over a 24 h ACRE-DEP exposure. Values represent the mean of n=3 independent experiments ± SD. *p ≤ .05. Figure S4. Epithelial cell MAPK inhibition pre-treatment with the ERK1/2 and p38 inhibitors, BVD-523 and SB203580, respectively. (A) mRNA expression of the antioxidants HMOX1, NQO1, and GCLM in endothelial cells after a 6 h ACRE-DEP exposure. (B) mRNA expression of IL-8 in endothelial cells after a 6 h ACRE-DEP exposure. (A-B) Values represent the mean of n=3 independent experiments ± SD. Statistically significant differences between VEH, ACRE-DEP, and DEP + ERKi/p38i cells are indicated by **p ≤ .01, ***p ≤ .001, and ****p ≤ .0001. Figure S5. (A) IL-1β, TNF-α and IL-6 secretion in the basolateral medium of the ACRE and ARE model after a 6 h ACRE-DEP exposure. Values represent the mean of n=3 independent experiments ± SD. ****p ≤ .0001. Figure S6. (A) mRNA expression of HMOX1, NQO1, GCLM and IL-8 expression in the epithelial cells after a 6 h ACRE-DEP exposure and epithelial cell MAPK inhibition pre-treatment. Values represent the mean of n=3 independent experiments ± SD. Statistically significant differences [file 12989_2024_576_MOESM3_ESM.docx]

**Supplemental Figures**

**Figure S1.**

**A.**

**
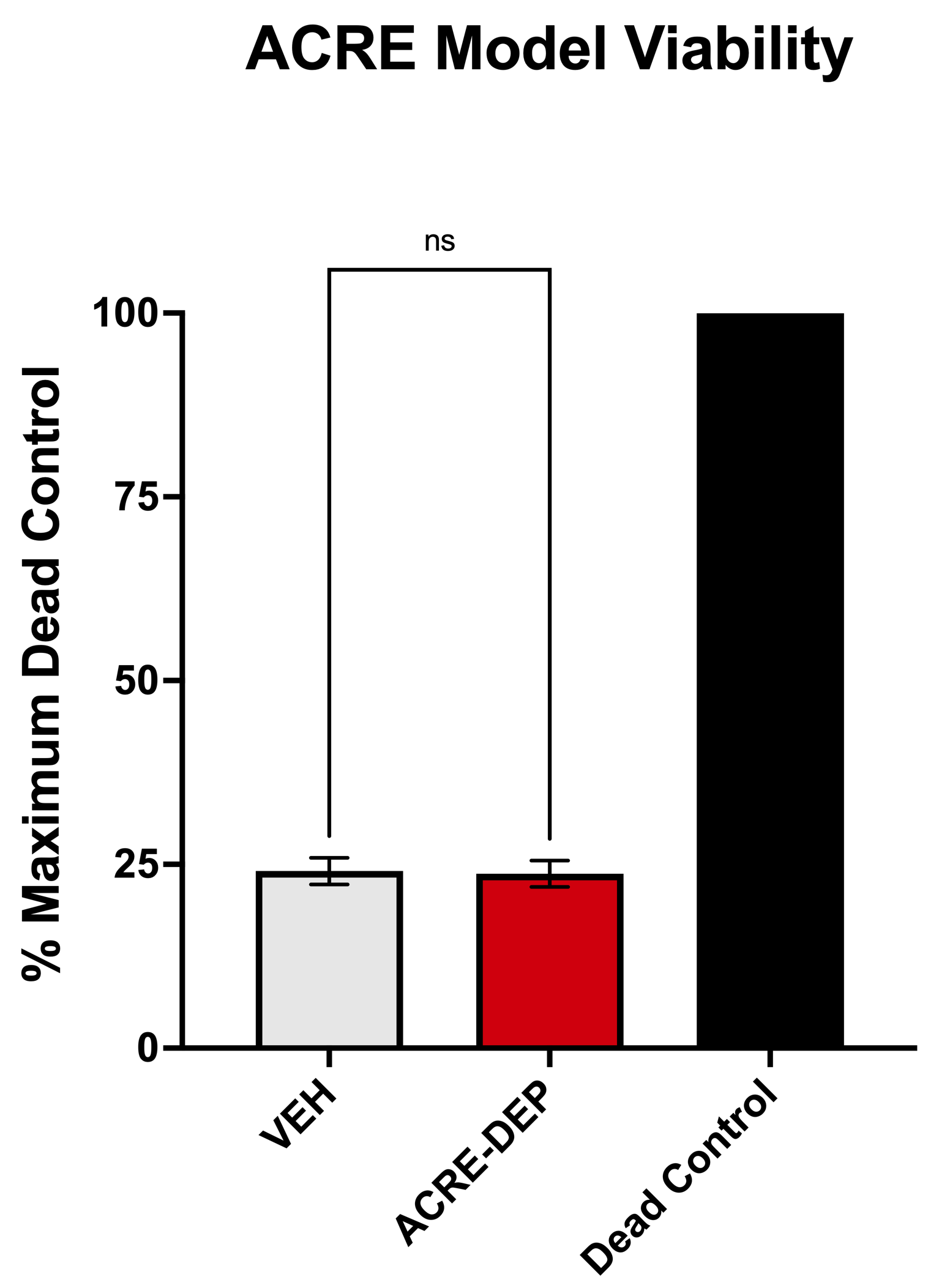
**

**Figure S2**


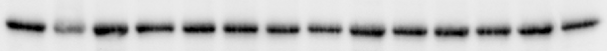

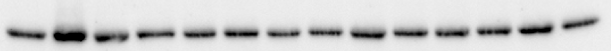

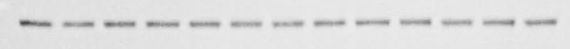

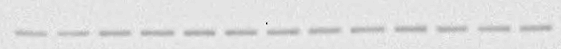


p-p65 (S536p)

p65

Vehicle (h)

PRE

2

2

4

4

6

6

8

8

10

10

24

24

DEP (h)

Vehicle (h)

PRE

2

2

4

4

6

6

8

8

10

10

24

24

DEP (h)

**B.**

**A.**


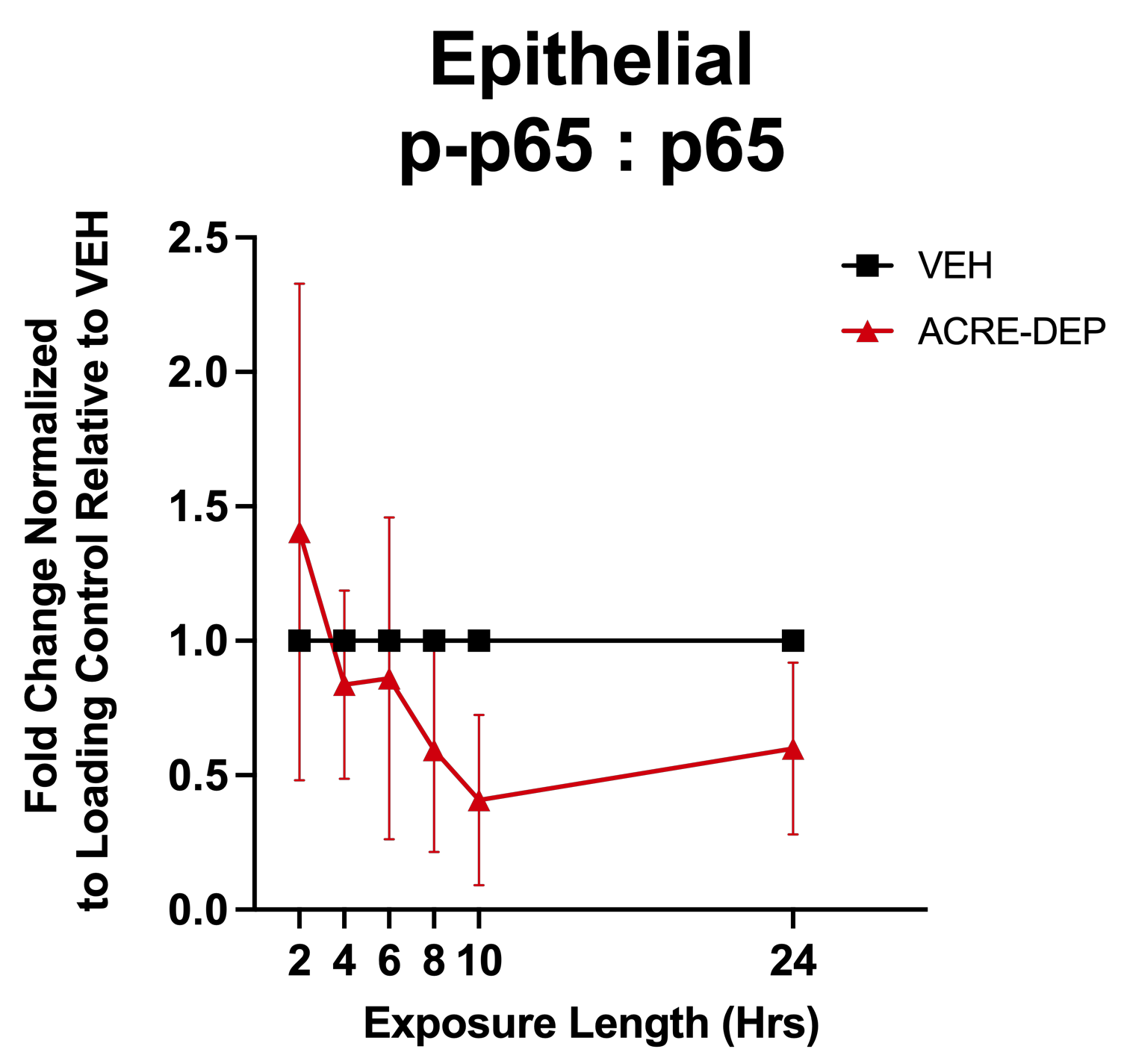

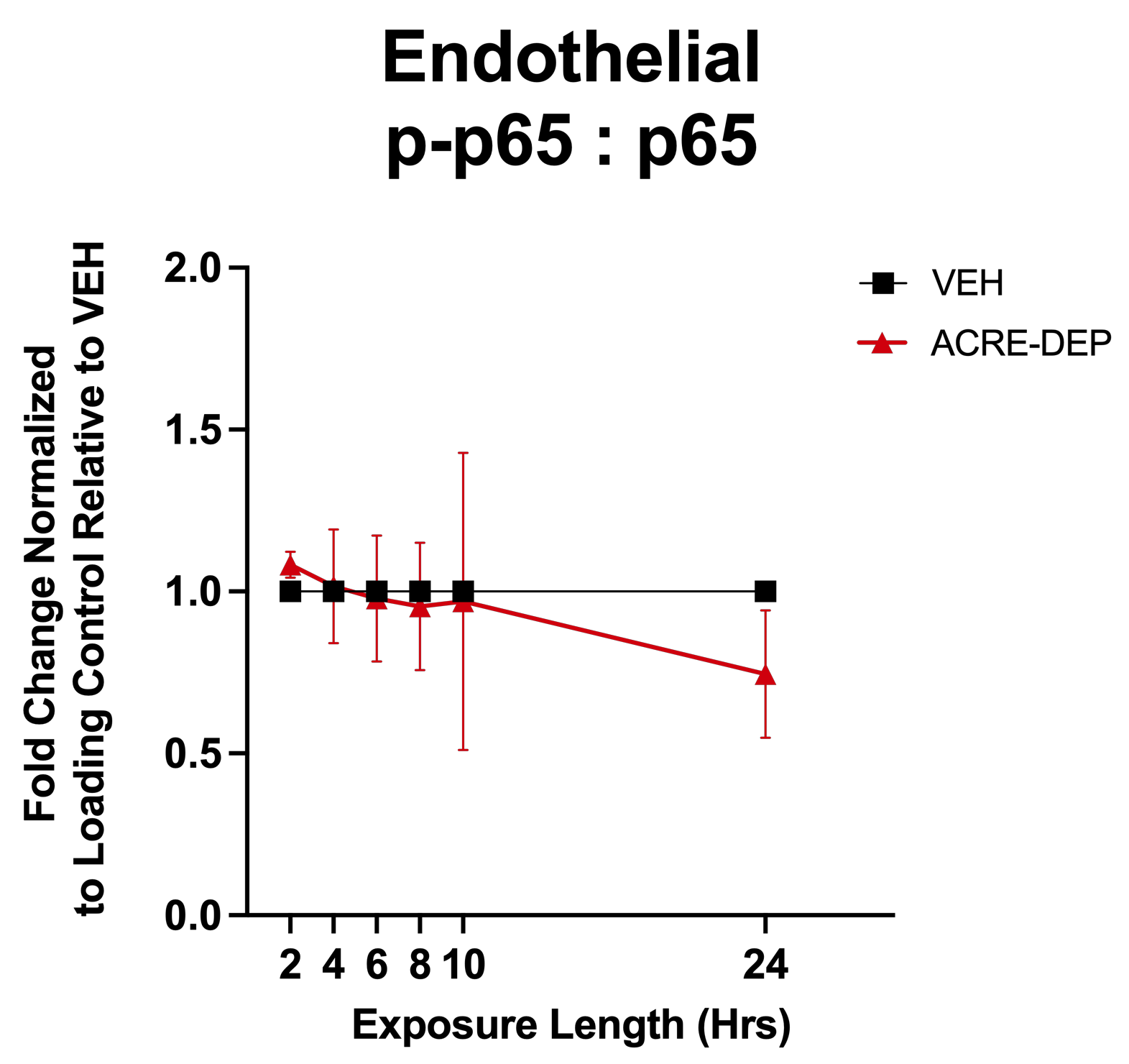


**Figure S3**

**A.**

**
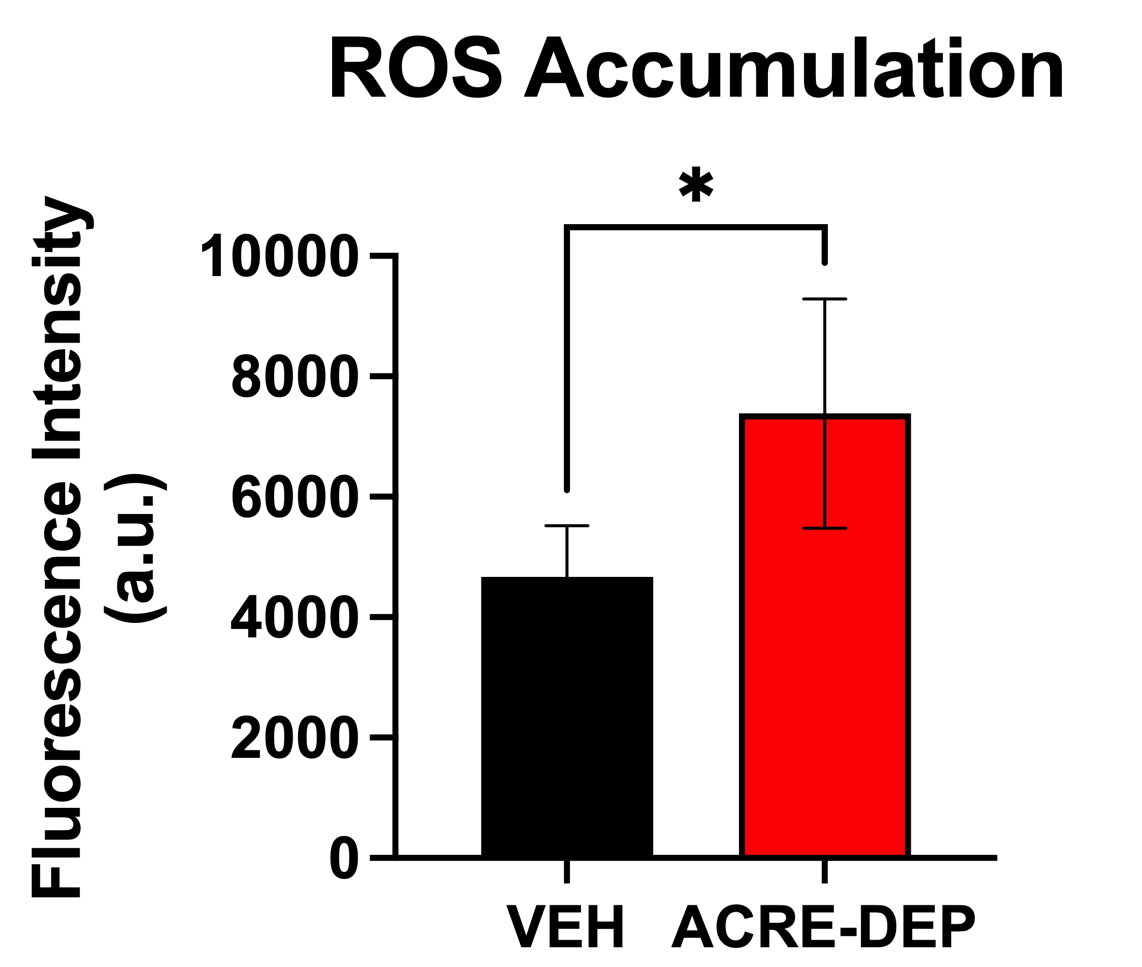
**

**Figure S4**

A

B


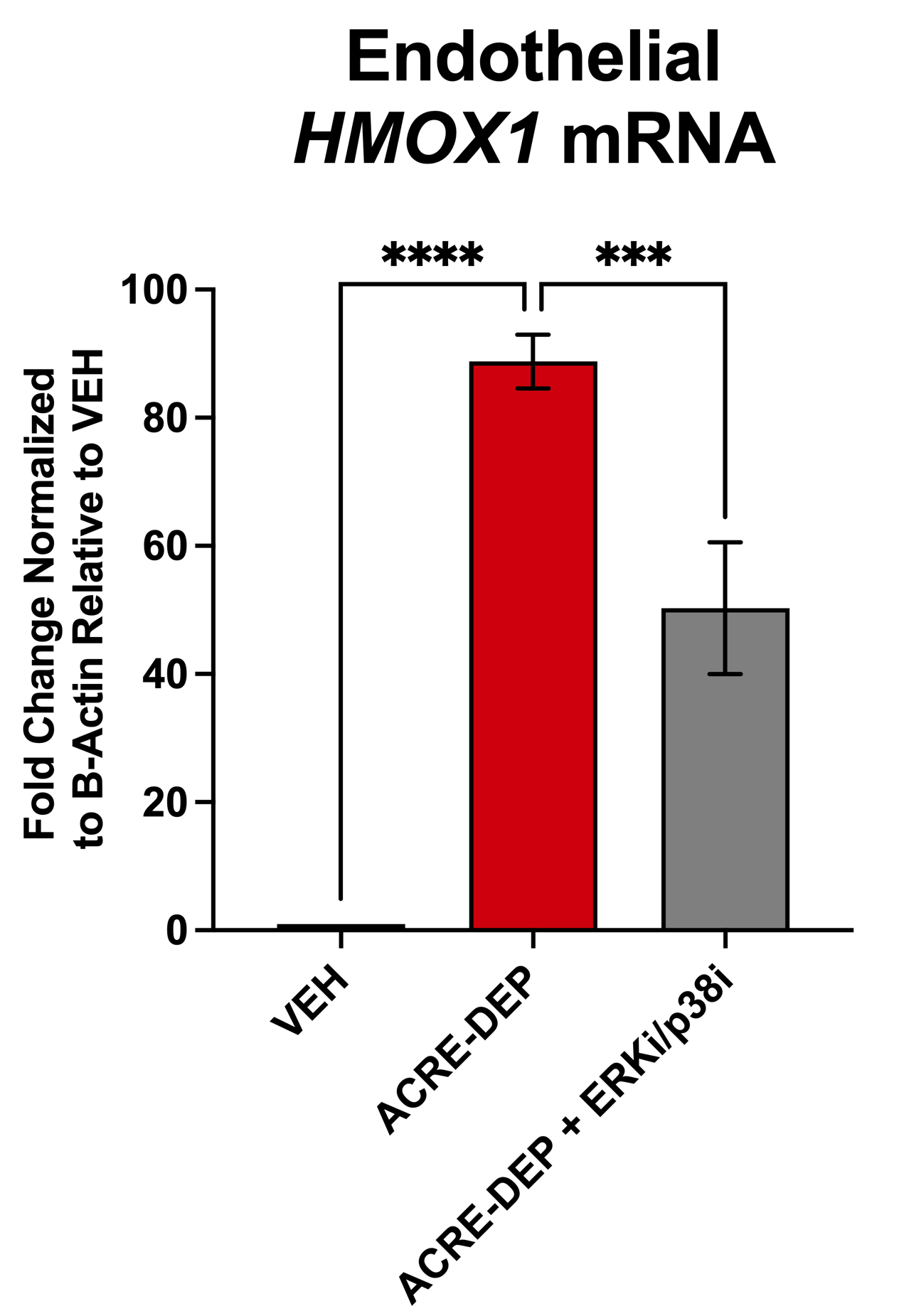

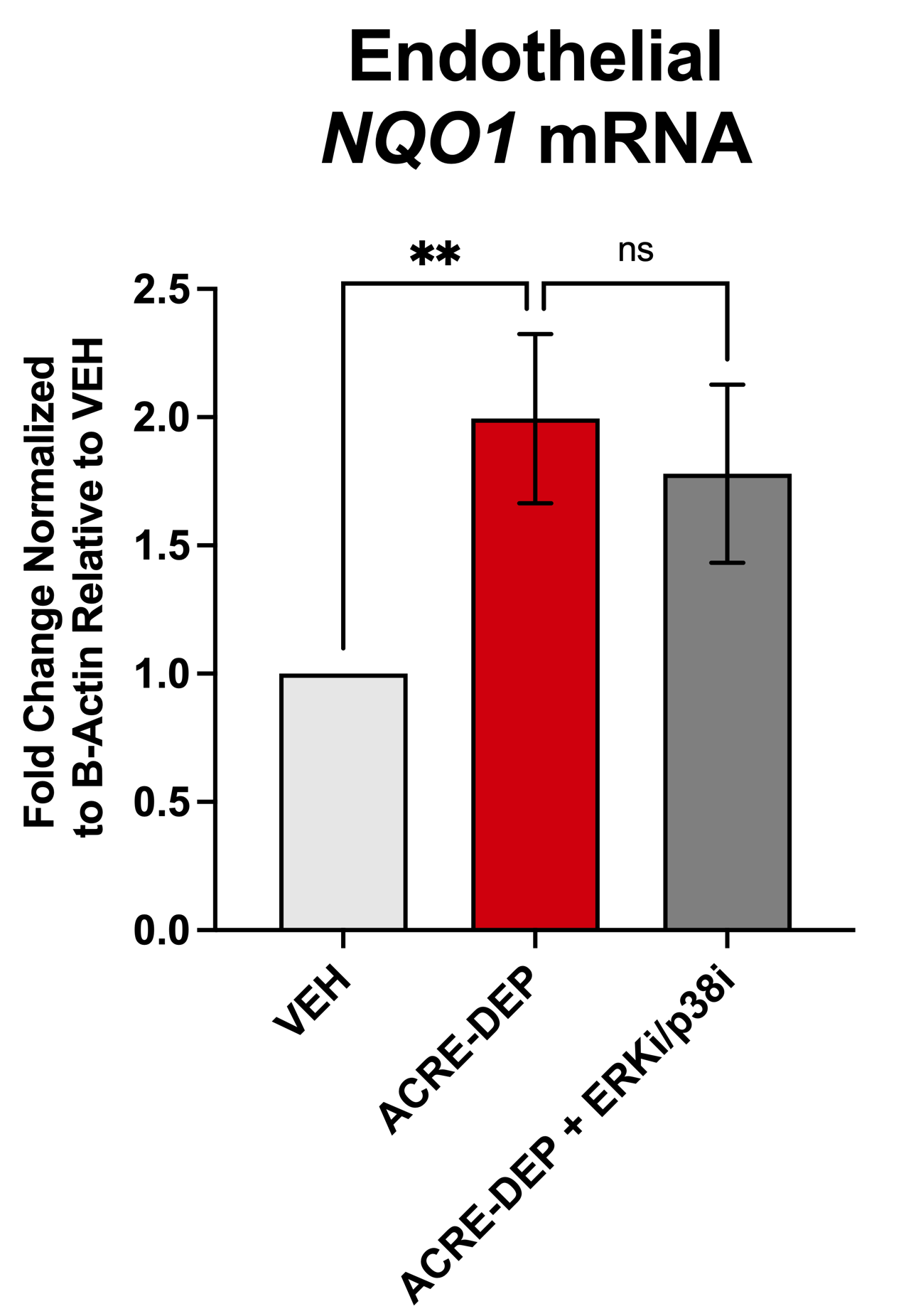

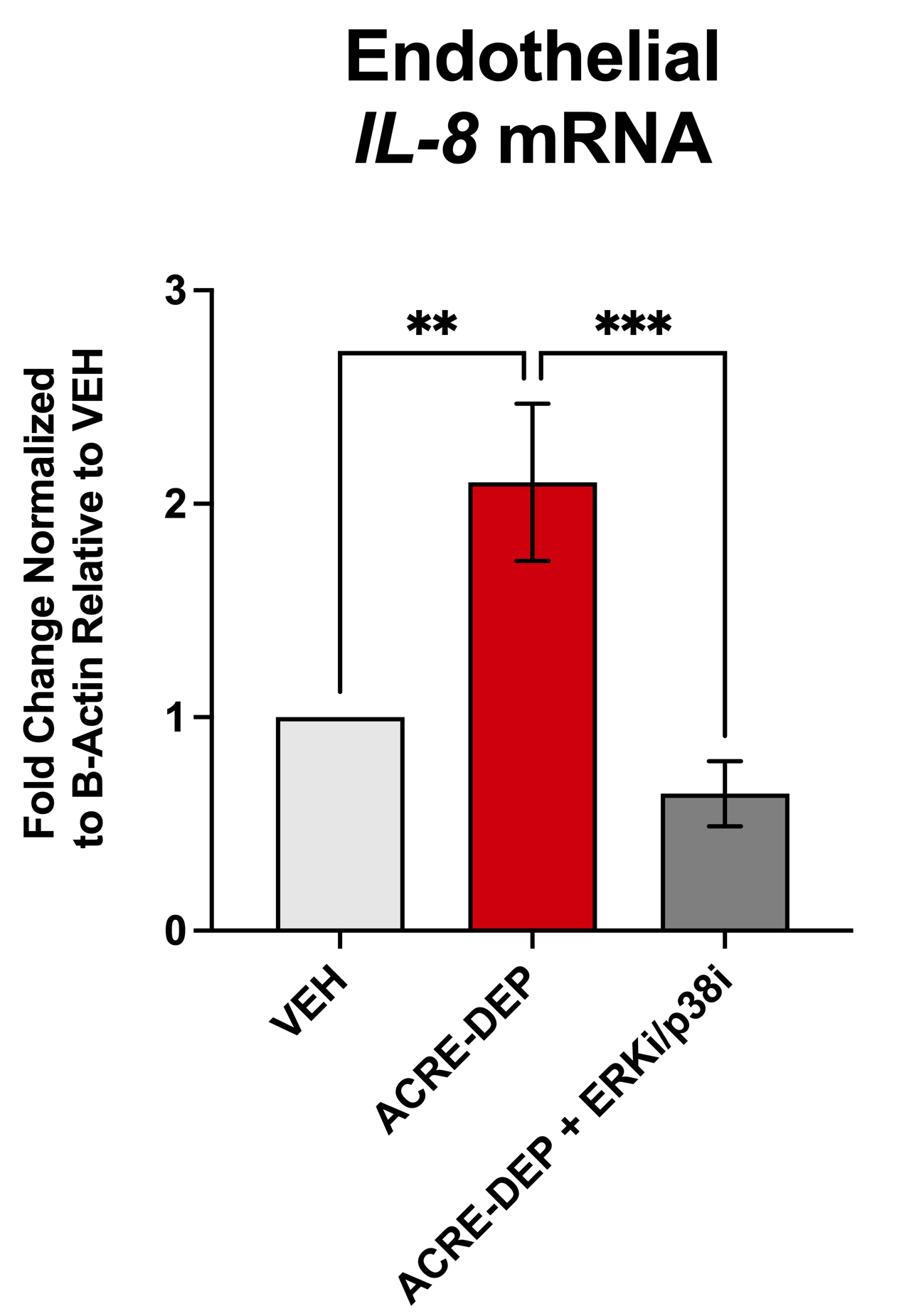

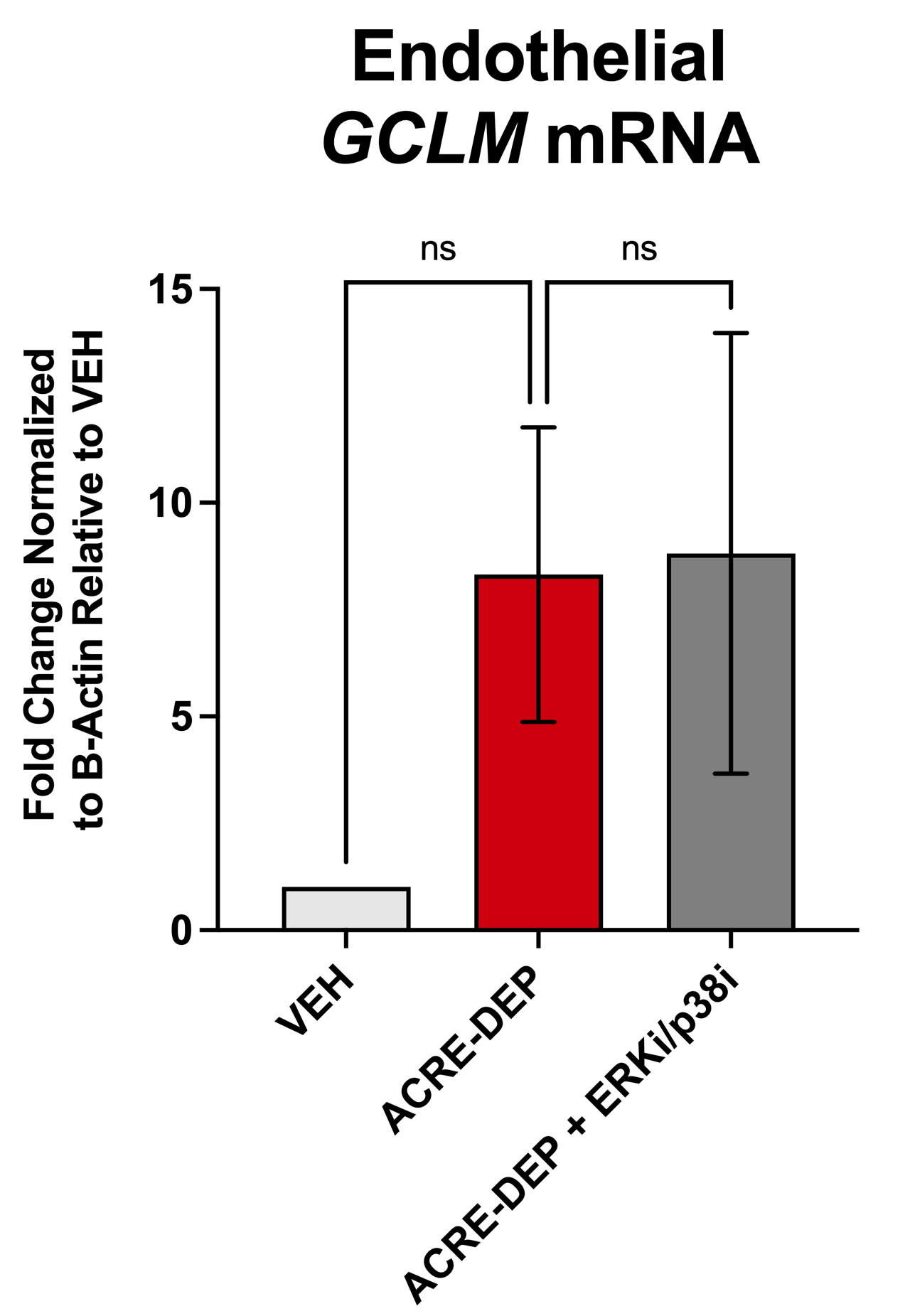


**Figure S5**

**A.**


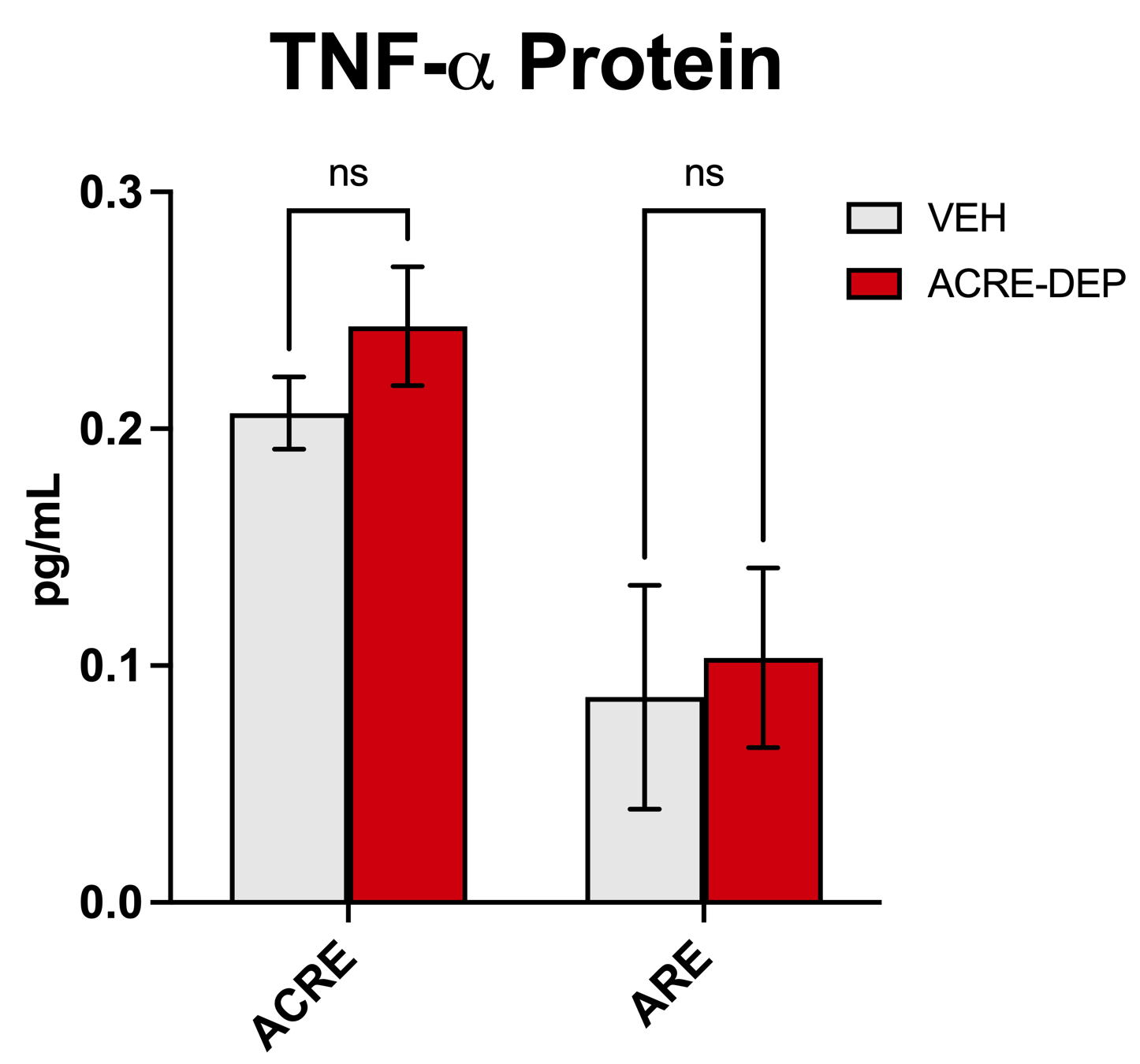

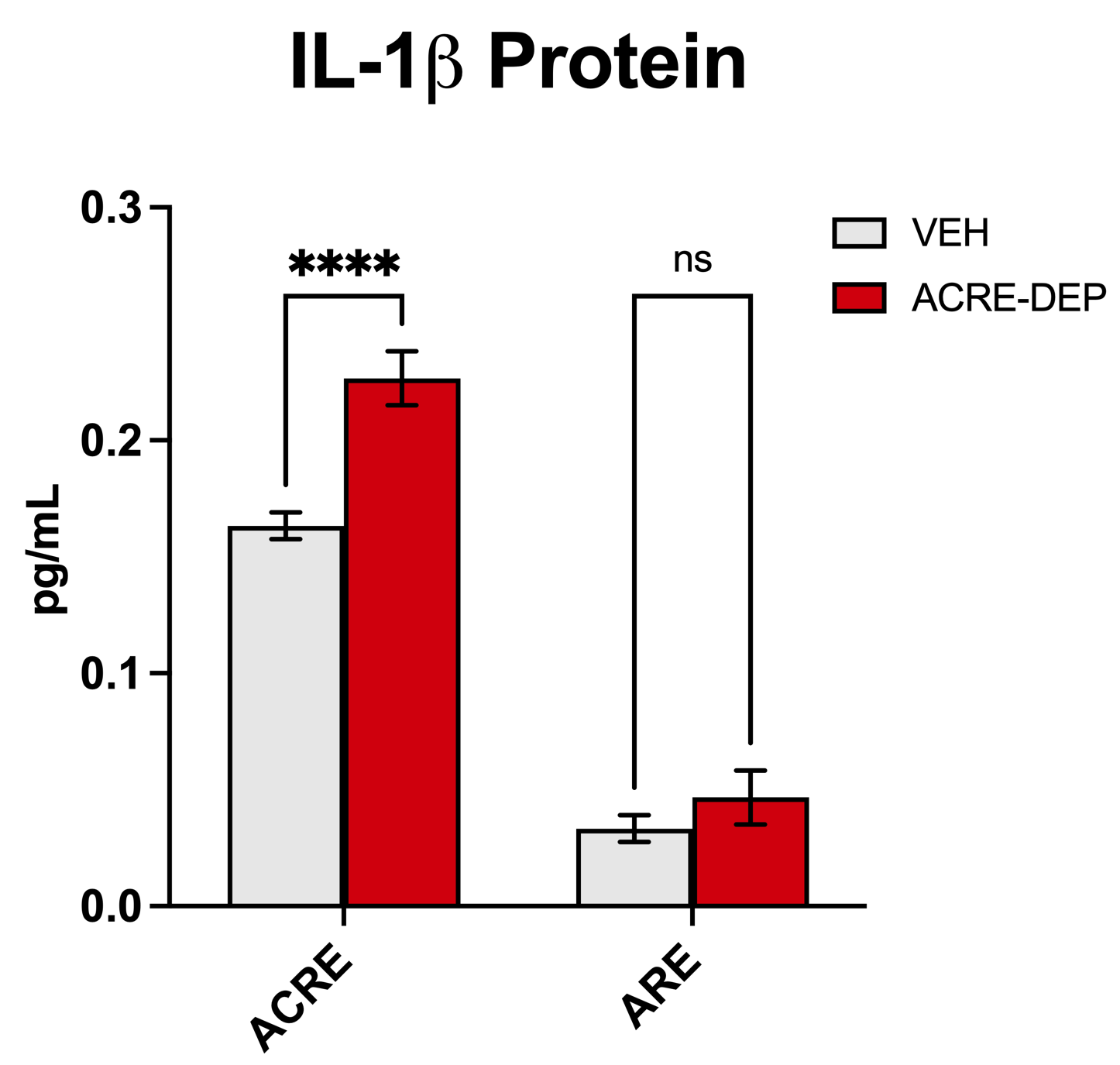

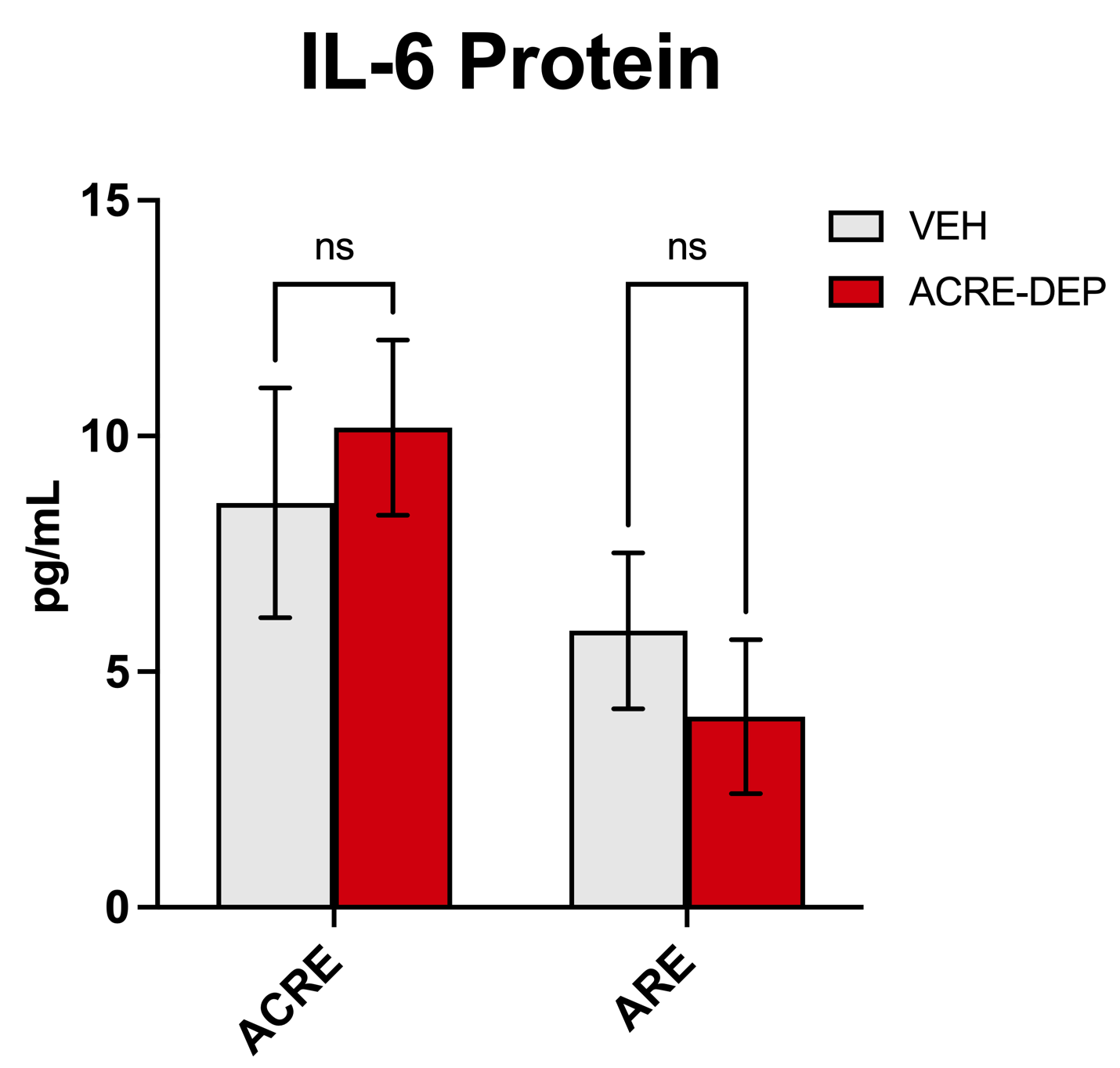


**Figure S6**

**A.**


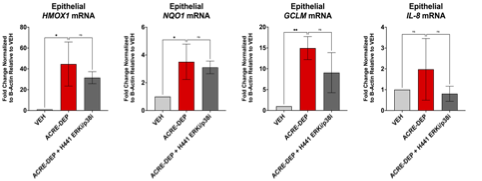


**Figure S7**

**A.**


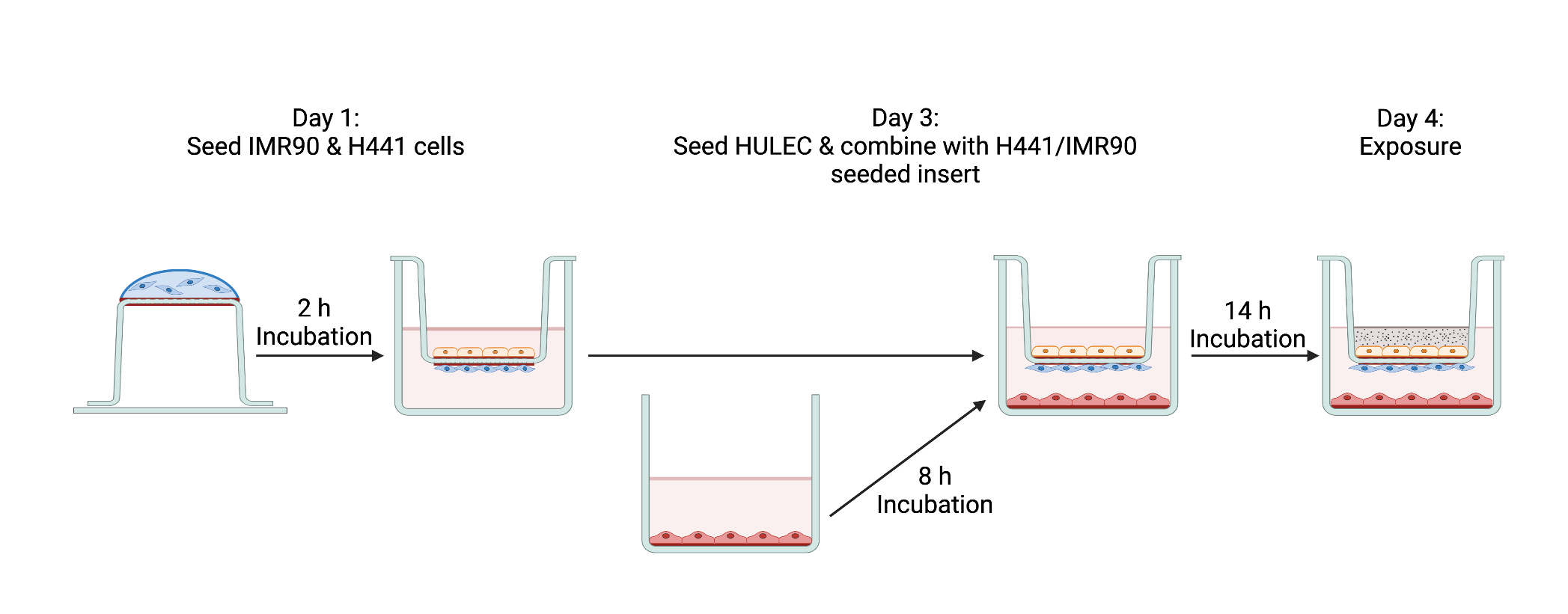


Seed HULEC

Seed H441

Seed IMR90
