# Supplementary material for: Epithelial MAPK signaling directs endothelial NRF2 signaling and IL-8 secretion in a tri-culture model of the alveolar-microvascular interface following diesel exhaust particulate (DEP) exposure
Source: Part Fibre Toxicol. 2024 Mar 11;21:15. doi: 10.1186/s12989-024-00576-8 (PMC10926573; doi:10.1186/s12989-024-00576-8)
Supplement: Supplementary file 4 — Additional file 4. Figure S8. Full length blots of all western blot images used in the manuscript. [file 12989_2024_576_MOESM4_ESM.pdf]

Fig S8A.

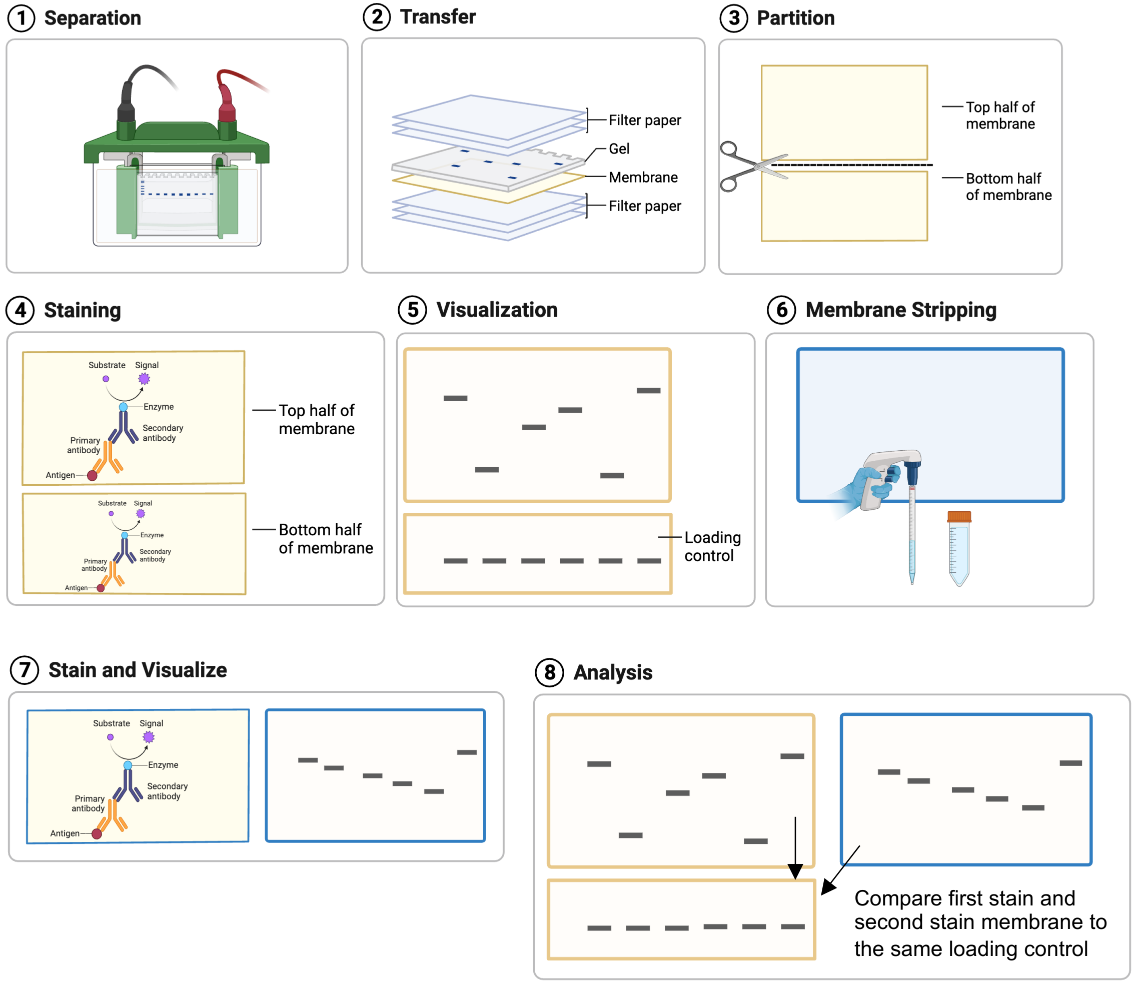

Fig. S8. **A.** Infographic depicting the western blotting strategy employed within the manuscript. In brief, the nitrocellulose membrane was cut into sections to maximize protein target evaluation. Furthermore, specific membranes could be stripped and re-probed for an additional protein. Note, analysis of target protein expression from the first stain and second stain was then completed using the loading control signal from the same membrane. An example of blots analyzed in this manner are denoted by a "star" icon in B. and E. **B-O.** Time course western blotting results on the epithelial (H441) and endothelial (HULEC) cells after VEH or DEP exposure. Lanes that contained samples not used within this manuscript are denoted with "—". N = 3 individual experiments conducted for each protein target. Arrows indicate the position of each specific protein. Cropped versions of western blots are shown in Fig. 4 – 6.

Fig S8B.

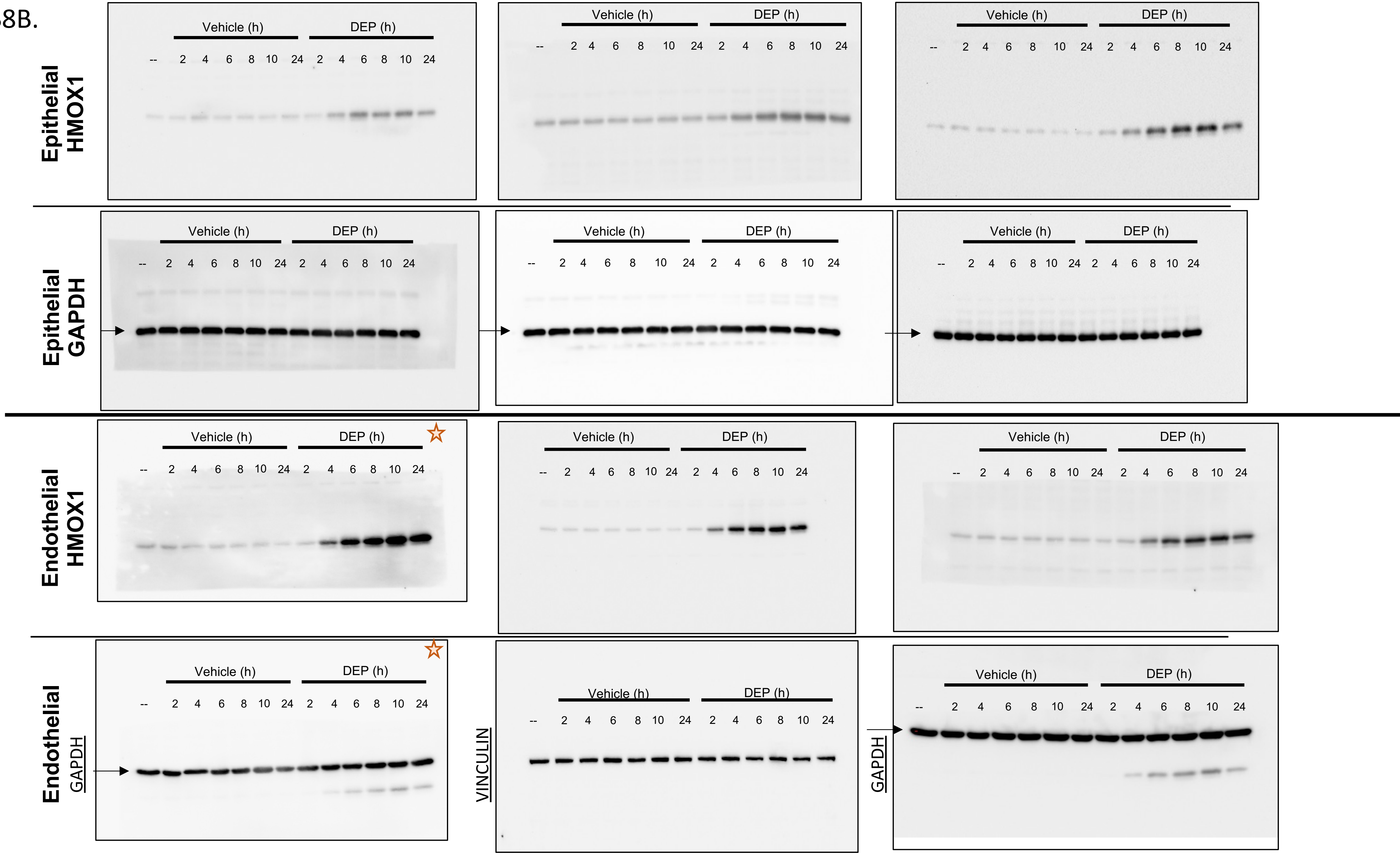

Epithelial HMOX1
Epithelial GAPDH
Endothelial HMOX1
Endothelial GAPDH
VINCULIN
GAPDH
Vehicle (h)
DEP (h)
-- 2 4 6 8 10 24 2 4 6 8 10 24

Fig S8C.

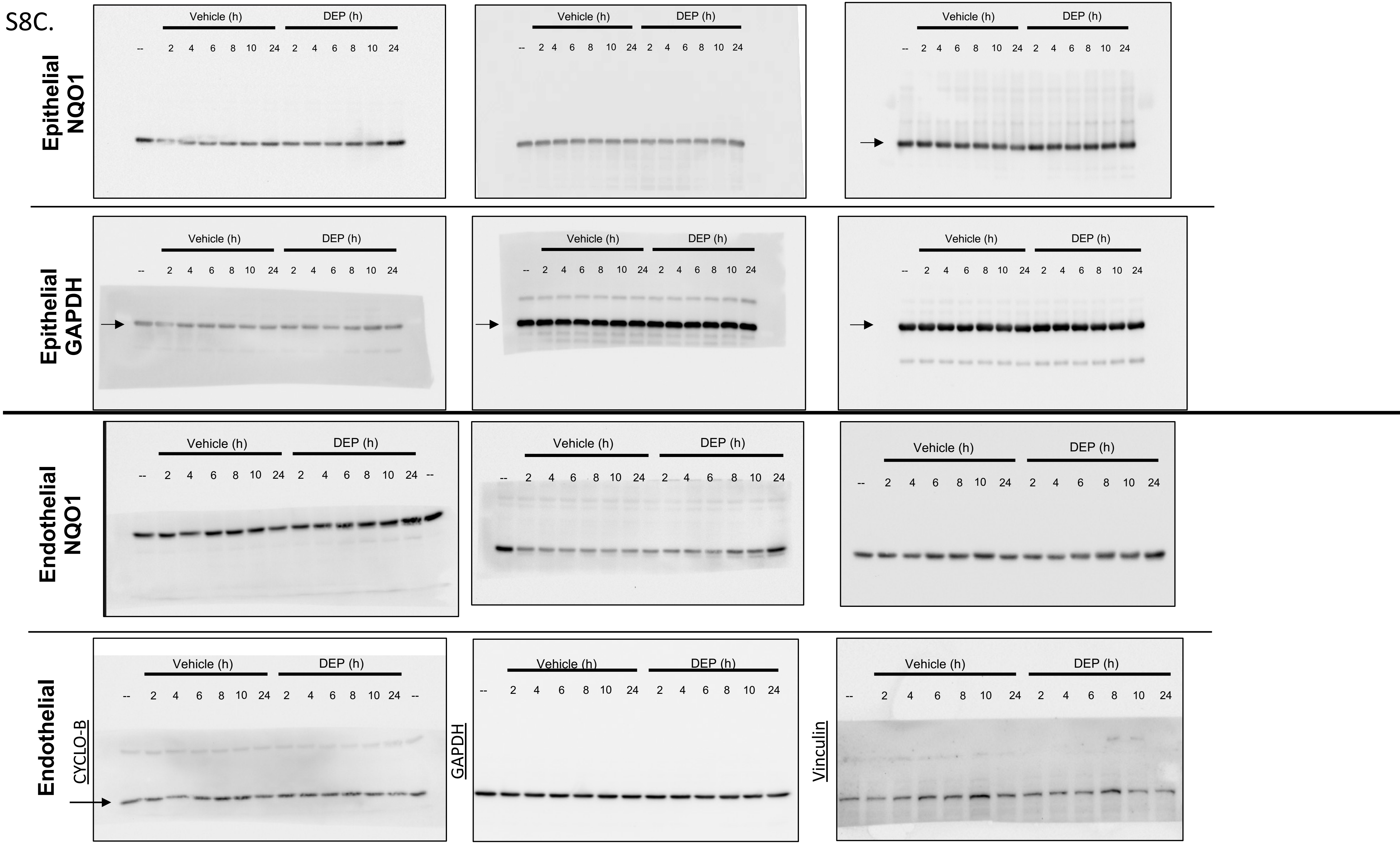
Epithelial NQO1
Vehicle (h)
DEP (h)
-- 2 4 6 8 10 24 2 4 6 8 10 24
Epithelial GAPDH
Vehicle (h)
DEP (h)
-- 2 4 6 8 10 24 2 4 6 8 10 24
Endothelial NQO1
Vehicle (h)
DEP (h)
-- 2 4 6 8 10 24 2 4 6 8 10 24 --
Endothelial
CYCLO-B
GAPDH
Vinculin
Vehicle (h)
DEP (h)
-- 2 4 6 8 10 24 2 4 6 8 10 24 --

Fig S8D.

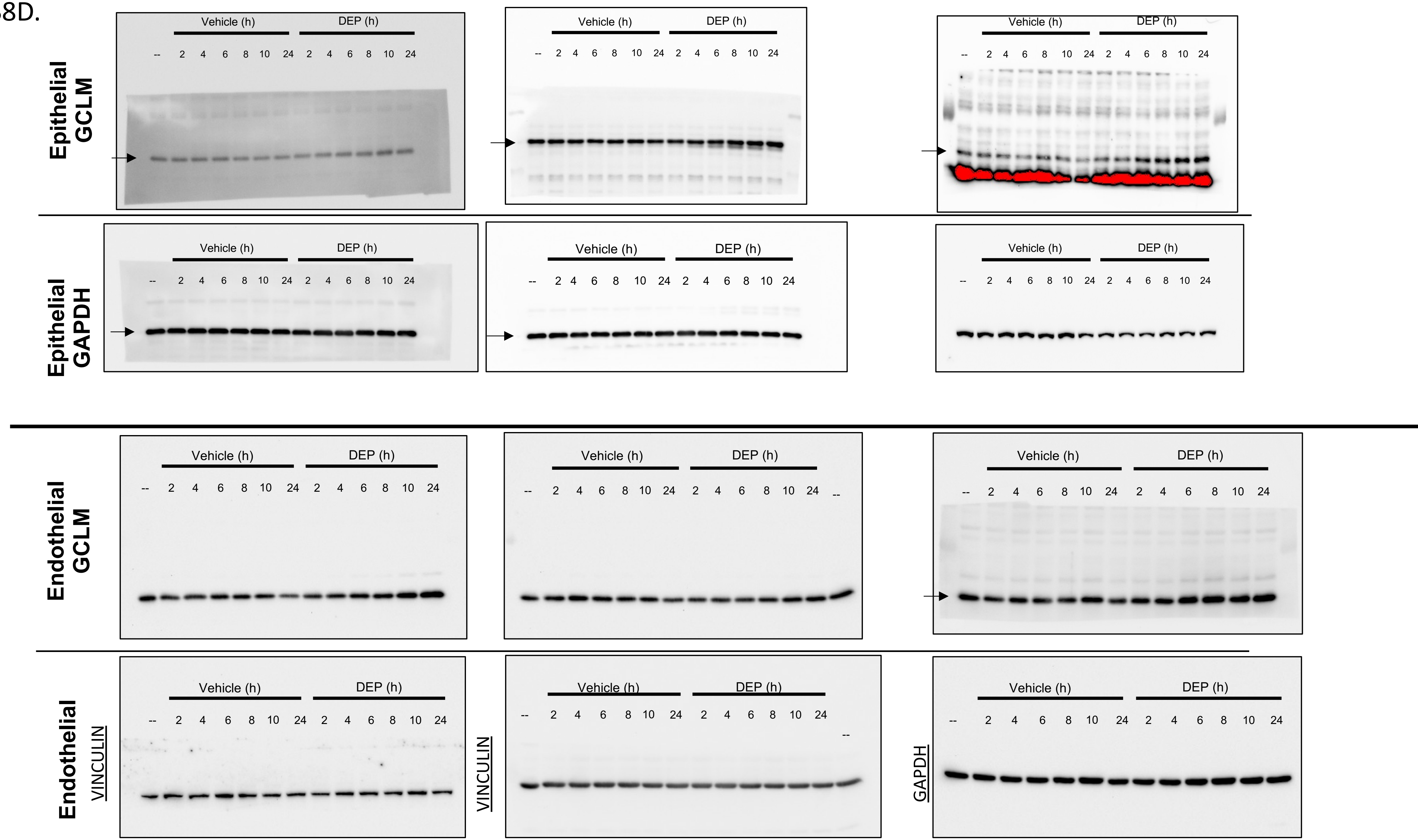
Epithelial GCLM
Epithelial GAPDH
Endothelial GCLM
Endothelial
VINCULIN
VINCULIN
GAPDH
Vehicle (h)
DEP (h)
-- 2 4 6 8 10 24 2 4 6 8 10 24

Fig S8E.

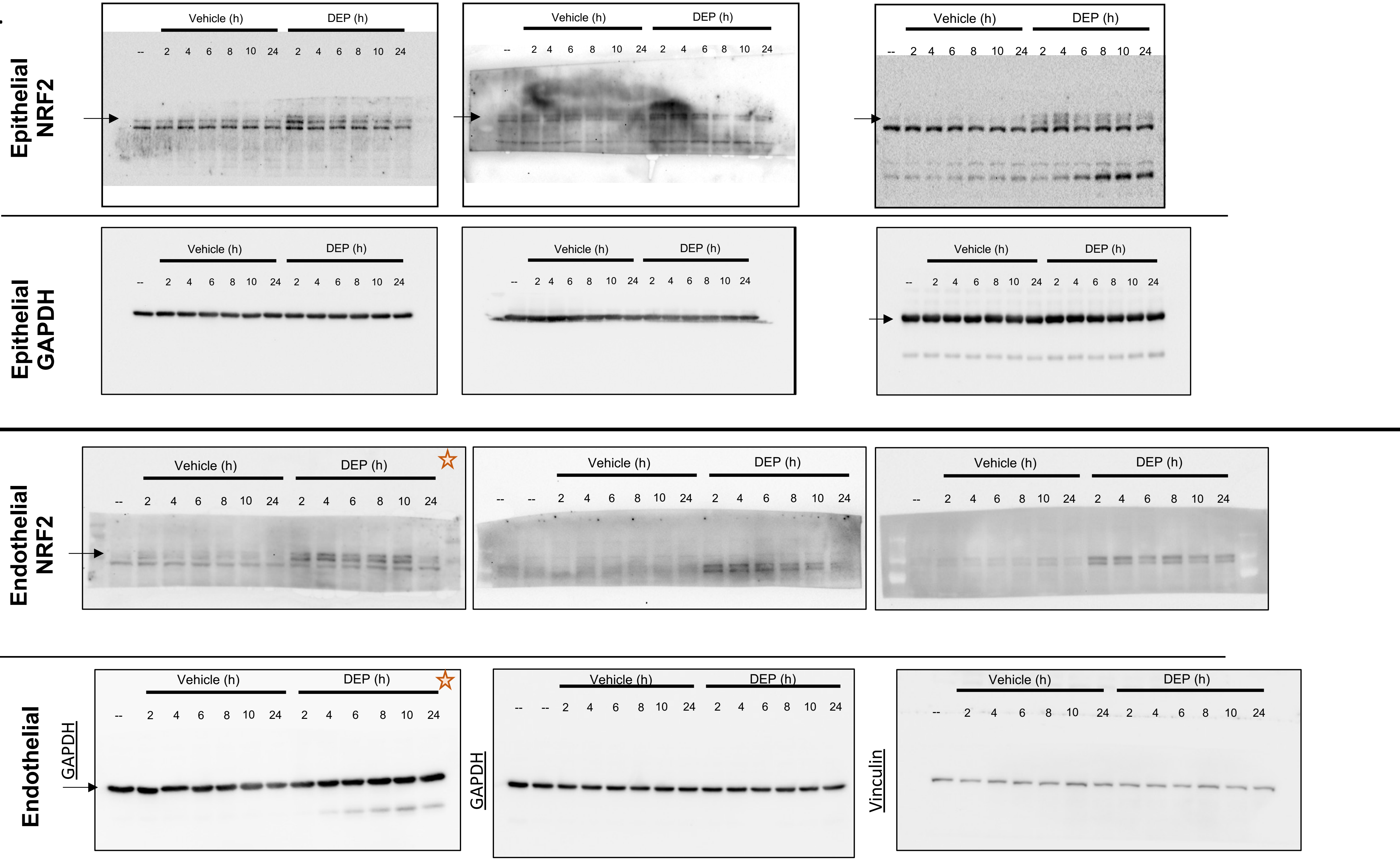
Epithelial NRF2
Epithelial GAPDH
Endothelial NRF2
Endothelial
GAPDH
Vinculin
Vehicle (h)
DEP (h)
-- 2 4 6 8 10 24 2 4 6 8 10 24

Fig S8F.

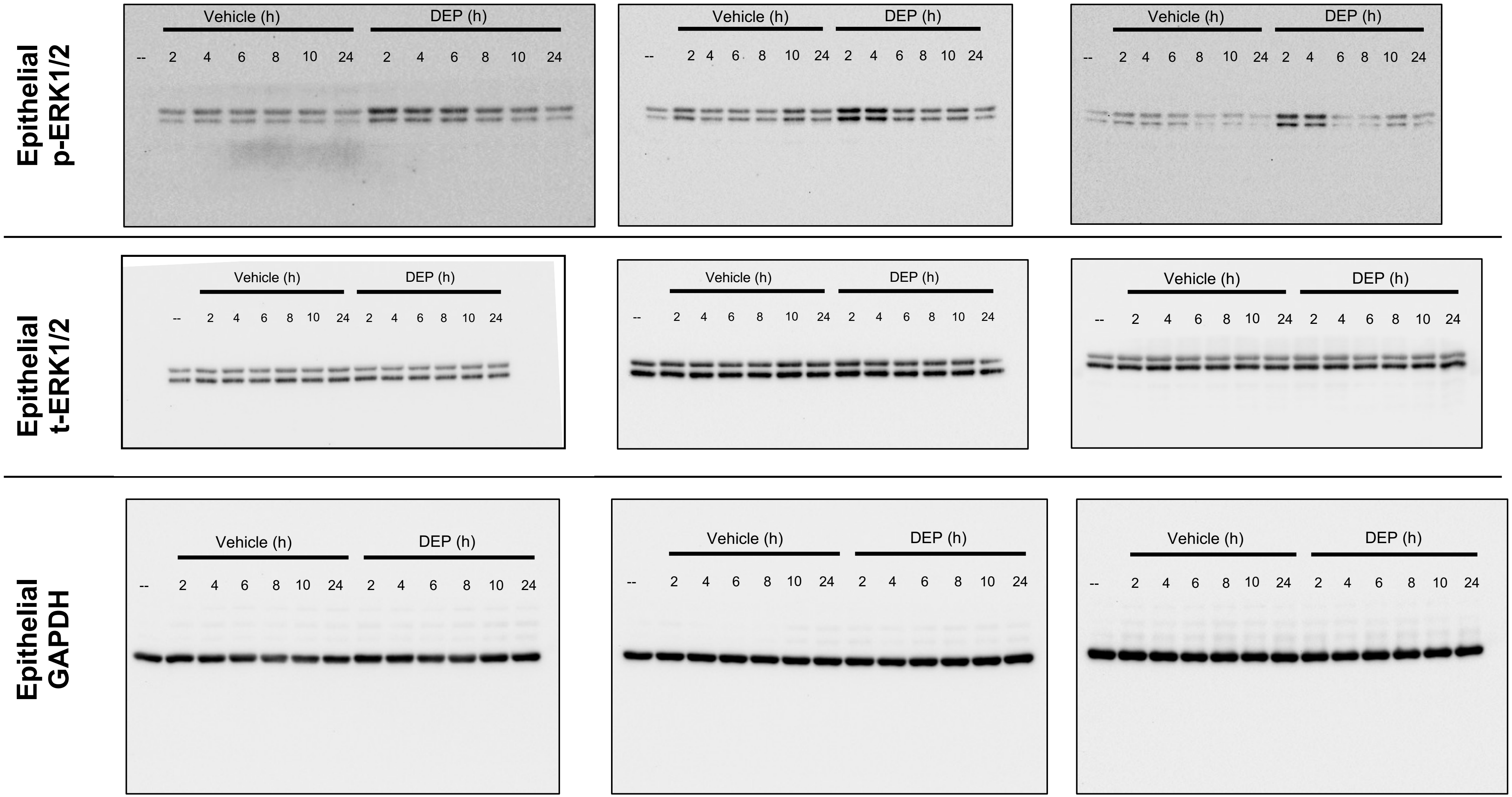
Epithelial p-ERK1/2
Epithelial t-ERK1/2
Epithelial GAPDH
Vehicle (h)
DEP (h)
-- 2 4 6 8 10 24 2 4 6 8 10 24

Fig S8G.

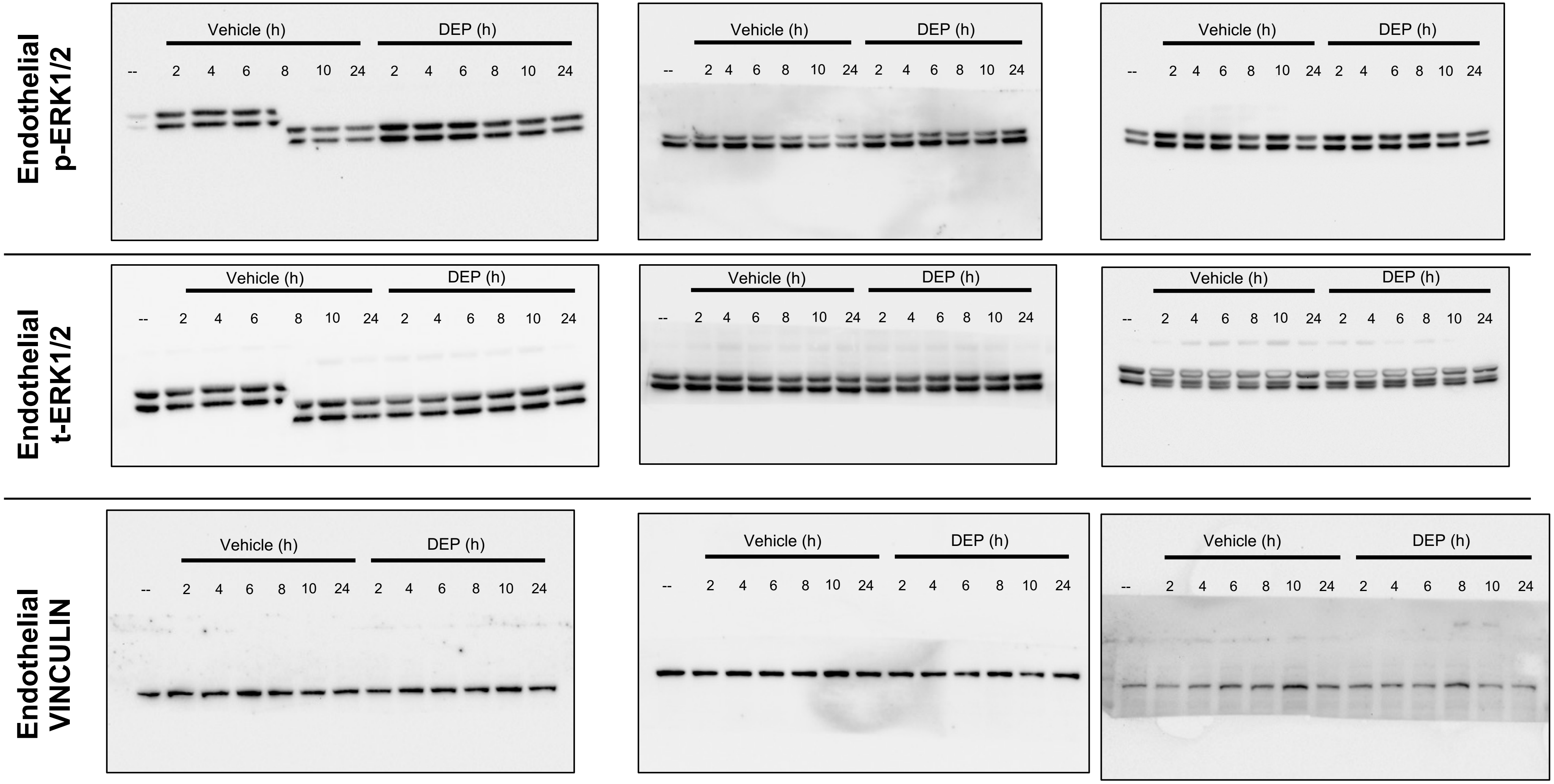
Endothelial p-ERK1/2
Endothelial t-ERK1/2
Endothelial VINCULIN
Vehicle (h)
DEP (h)
-- 2 4 6 8 10 24 2 4 6 8 10 24

Fig S8H.

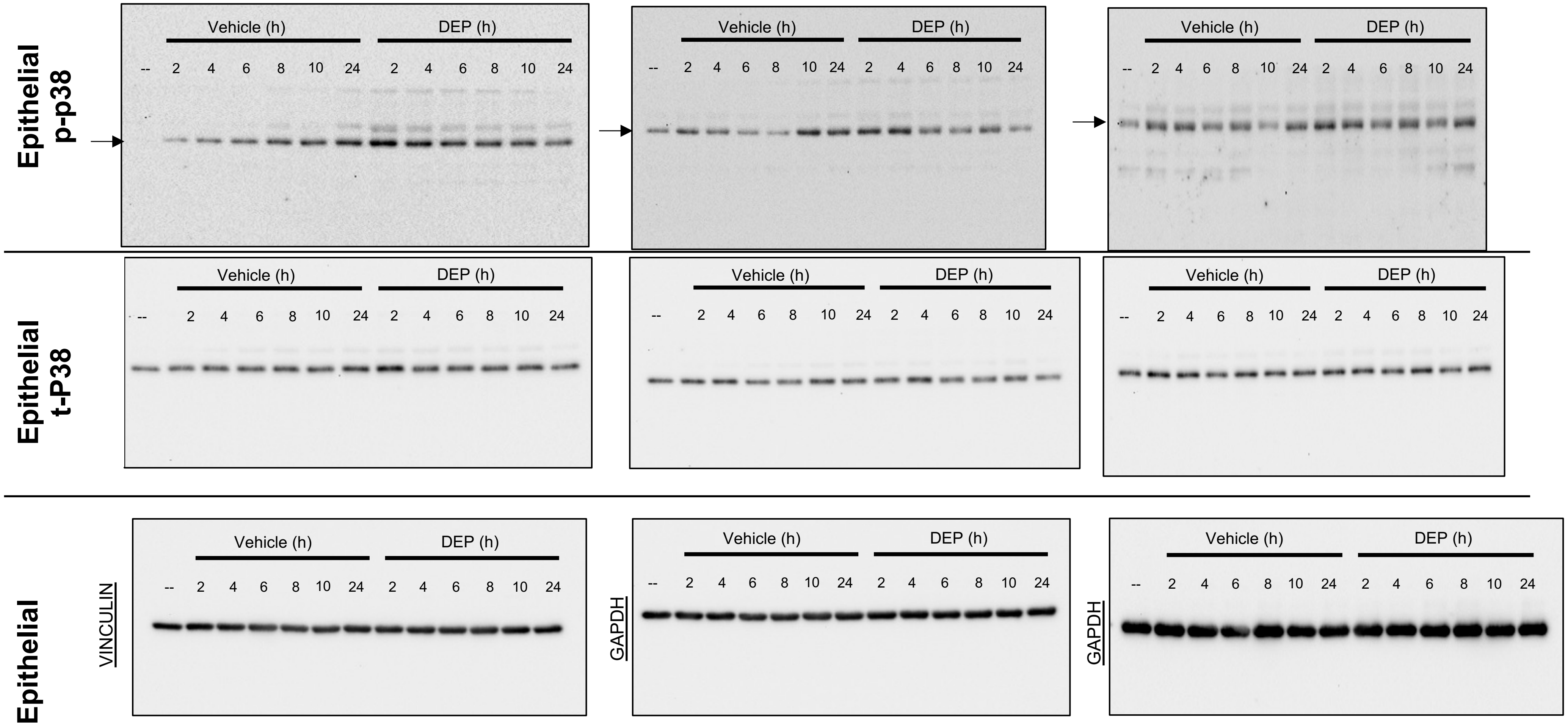
Epithelial p-p38
Epithelial t-P38
Epithelial
Vehicle (h)
DEP (h)
-- 2 4 6 8 10 24 2 4 6 8 10 24
VINCULIN
GAPDH

Fig S8I.

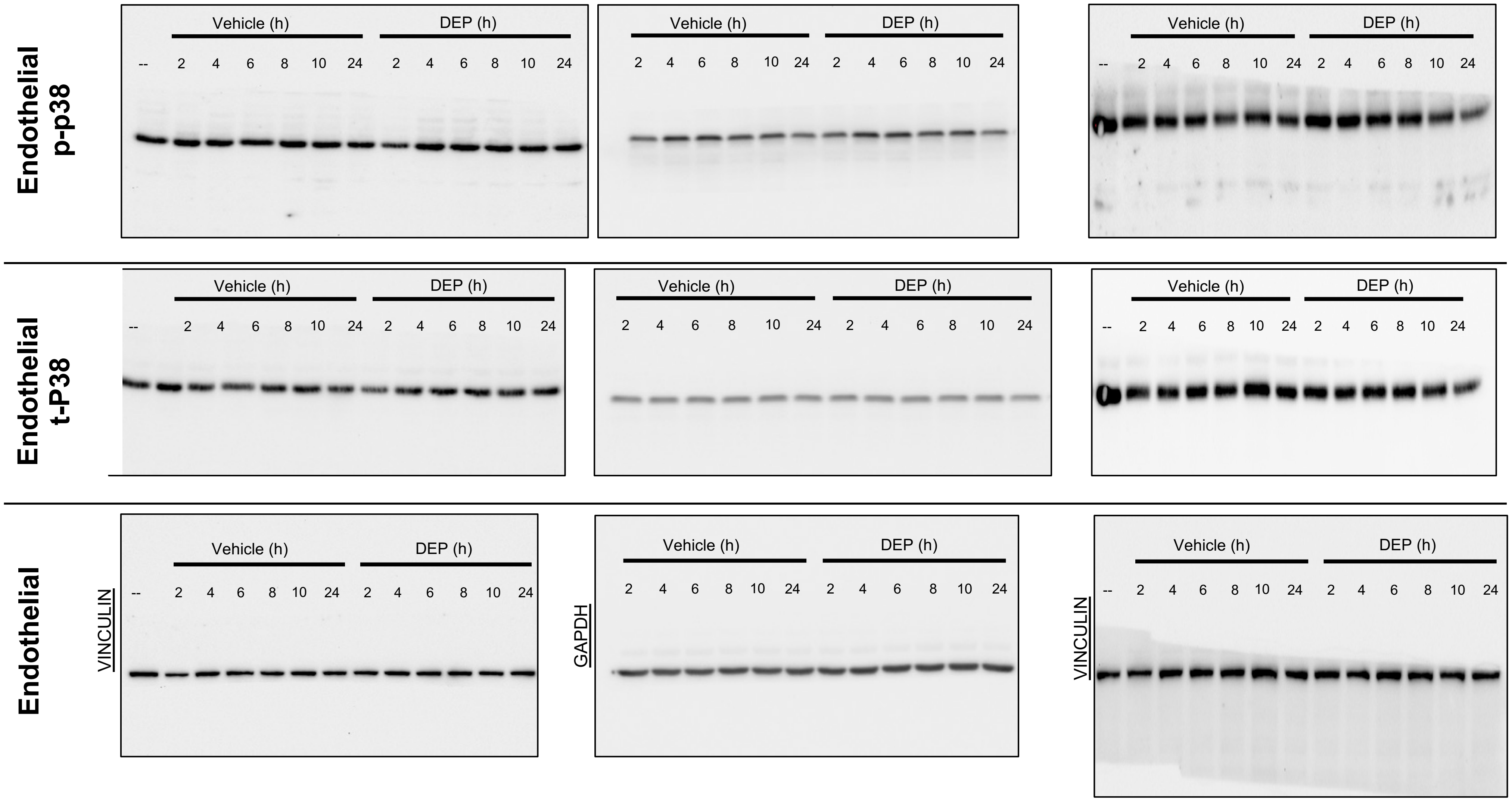
Endothelial
p-p38
Vehicle (h)
DEP (h)
-- 2 4 6 8 10 24 2 4 6 8 10 24
Vehicle (h)
DEP (h)
2 4 6 8 10 24 2 4 6 8 10 24
Vehicle (h)
DEP (h)
-- 2 4 6 8 10 24 2 4 6 8 10 24
Endothelial
t-P38
Vehicle (h)
DEP (h)
-- 2 4 6 8 10 24 2 4 6 8 10 24
Vehicle (h)
DEP (h)
2 4 6 8 10 24 2 4 6 8 10 24
Vehicle (h)
DEP (h)
-- 2 4 6 8 10 24 2 4 6 8 10 24
Endothelial
VINCULIN
Vehicle (h)
DEP (h)
-- 2 4 6 8 10 24 2 4 6 8 10 24
GAPDH
Vehicle (h)
DEP (h)
2 4 6 8 10 24 2 4 6 8 10 24
VINCULIN
Vehicle (h)
DEP (h)
-- 2 4 6 8 10 24 2 4 6 8 10 24

Fig S8J.

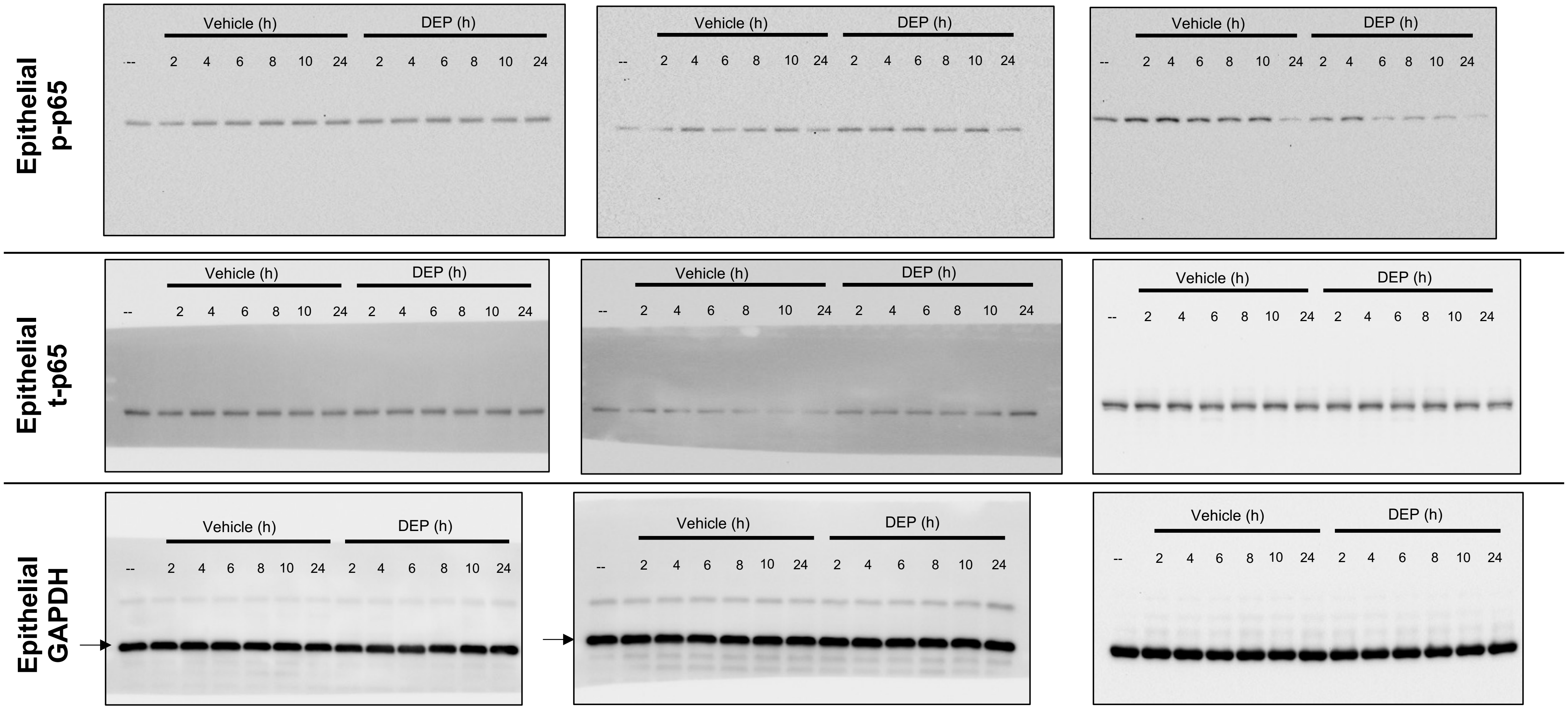
Epithelial p-p65
Epithelial t-p65
Epithelial GAPDH
Vehicle (h)
DEP (h)
-- 2 4 6 8 10 24 2 4 6 8 10 24

Fig S8K.

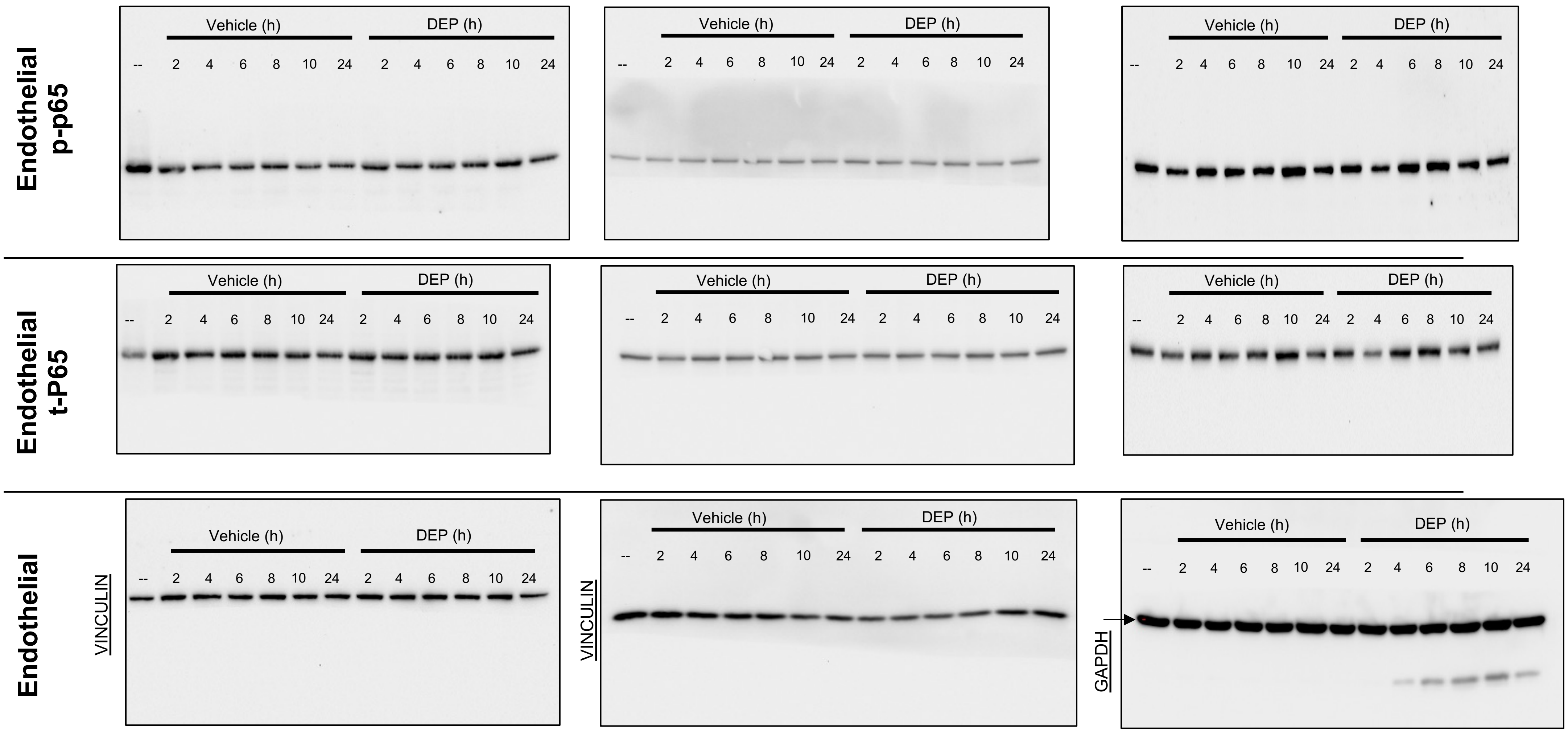
Endothelial
p-p65
Vehicle (h)
DEP (h)
-- 2 4 6 8 10 24 2 4 6 8 10 24
Endothelial
t-P65
Vehicle (h)
DEP (h)
-- 2 4 6 8 10 24 2 4 6 8 10 24
Endothelial
VINCULIN
Vehicle (h)
DEP (h)
-- 2 4 6 8 10 24 2 4 6 8 10 24
GAPDH

Fig S8L.

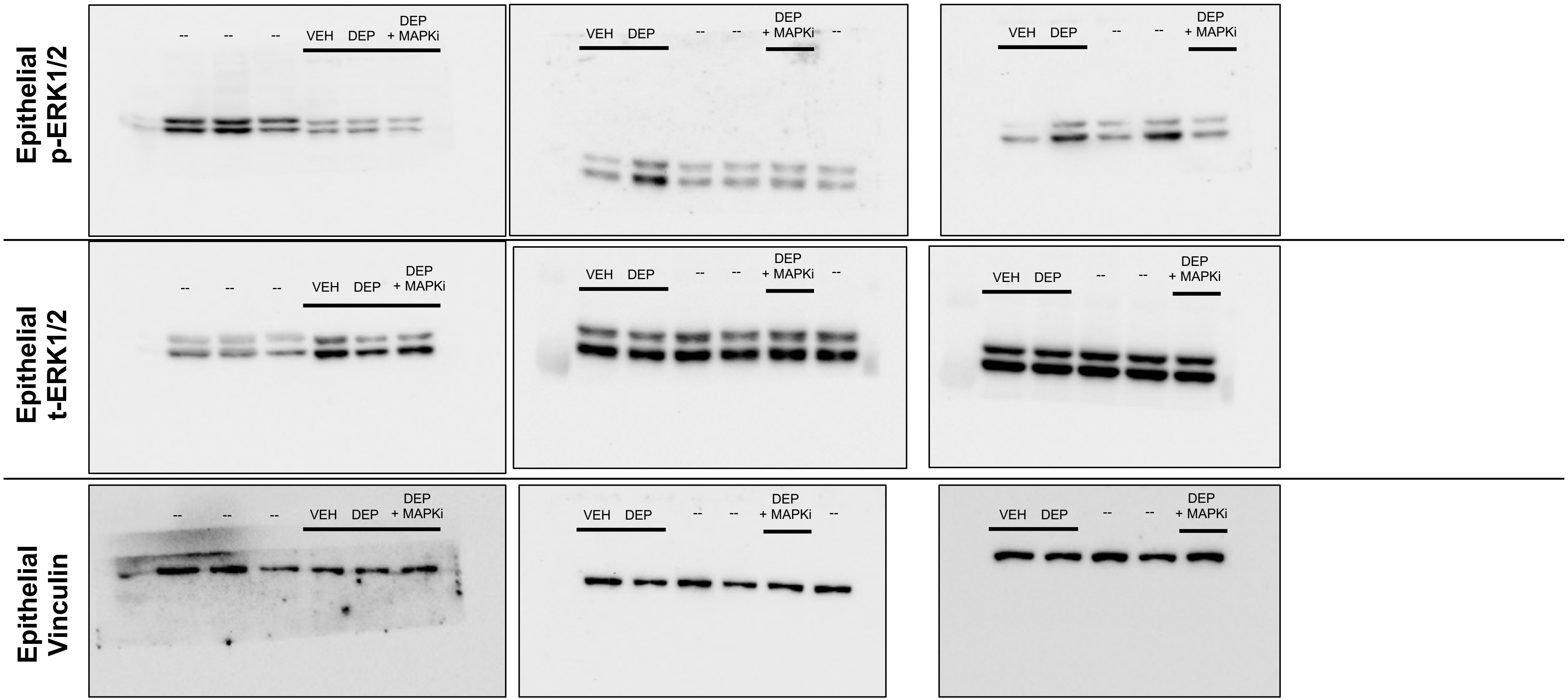
Epithelial
p-ERK1/2
-- -- -- VEH DEP DEP + MAPKi
VEH DEP -- -- DEP + MAPKi --
VEH DEP -- -- DEP + MAPKi
Epithelial
t-ERK1/2
-- -- -- VEH DEP DEP + MAPKi
VEH DEP -- -- DEP + MAPKi --
VEH DEP -- -- DEP + MAPKi
Epithelial
Vinculin
-- -- -- VEH DEP DEP + MAPKi
VEH DEP -- -- DEP + MAPKi --
VEH DEP -- -- DEP + MAPKi

Fig S8M.

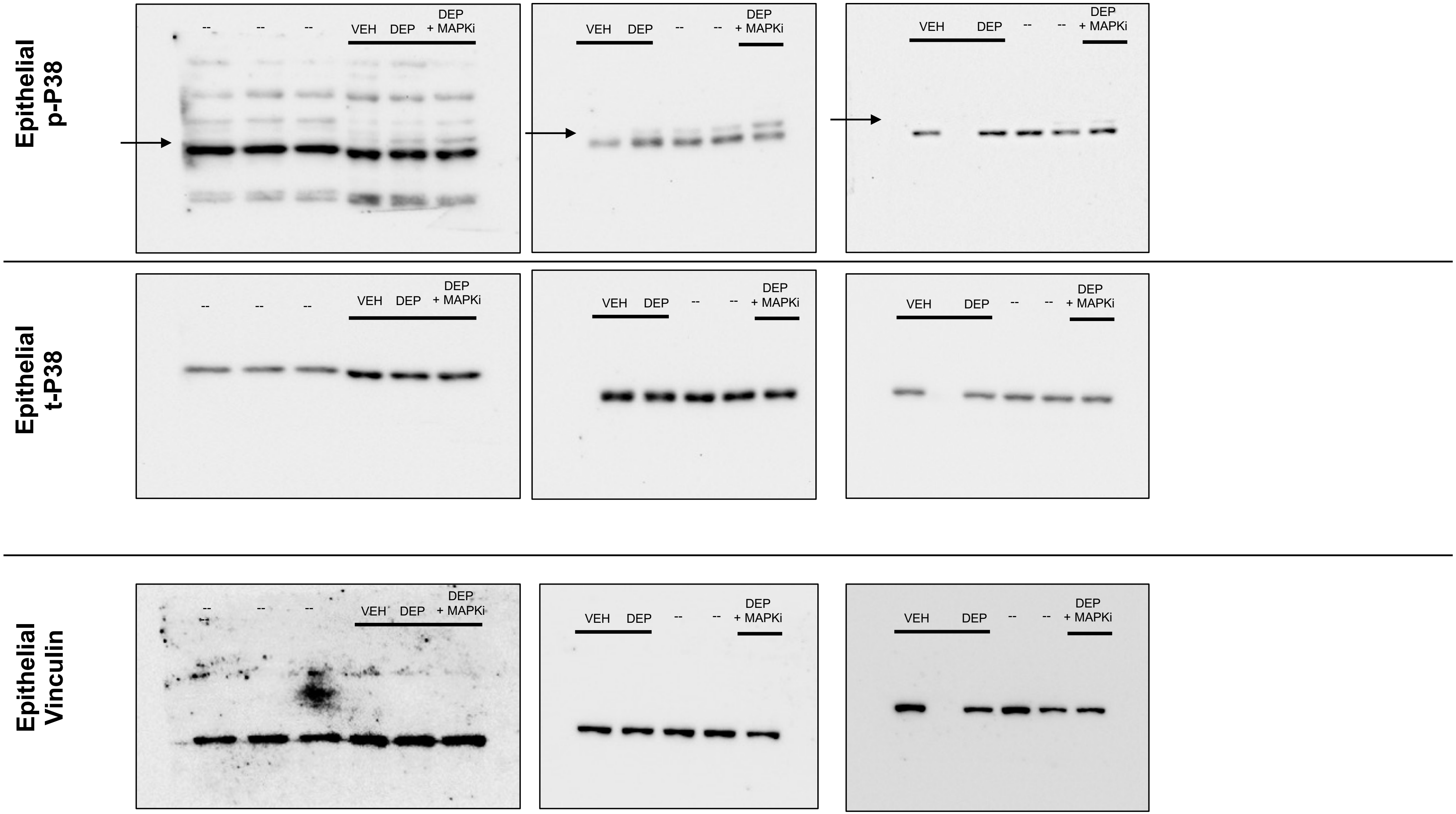
Epithelial p-P38
Epithelial t-P38
Epithelial Vinculin
--
VEH
DEP
DEP + MAPKi

Fig S8N.

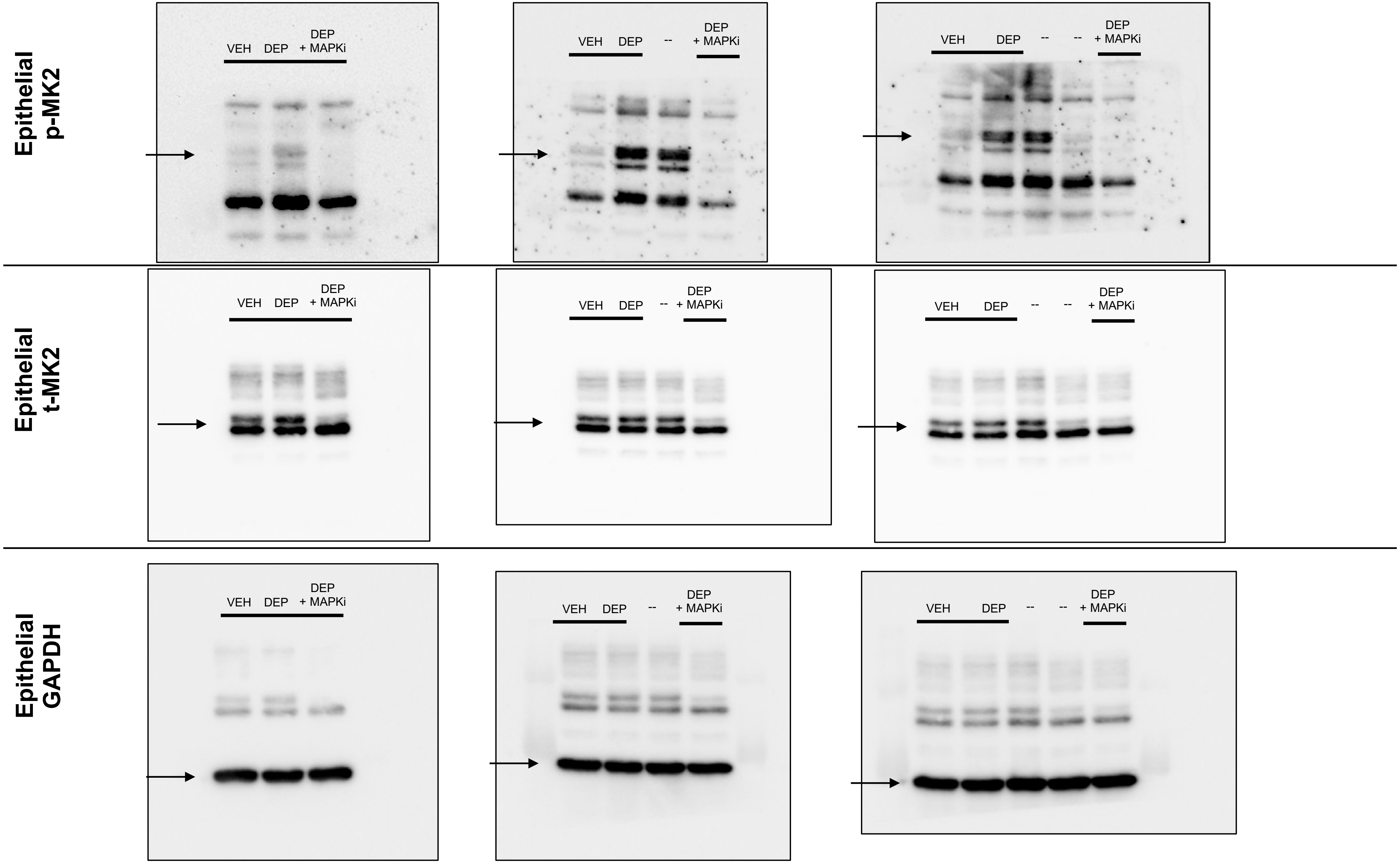
Epithelial p-MK2
VEH DEP DEP + MAPKi
VEH DEP -- DEP + MAPKi
VEH DEP -- -- DEP + MAPKi
Epithelial t-MK2
VEH DEP DEP + MAPKi
VEH DEP -- DEP + MAPKi
VEH DEP -- -- DEP + MAPKi
Epithelial GAPDH
VEH DEP DEP + MAPKi
VEH DEP -- DEP + MAPKi
VEH DEP -- -- DEP + MAPKi

Fig S8O.

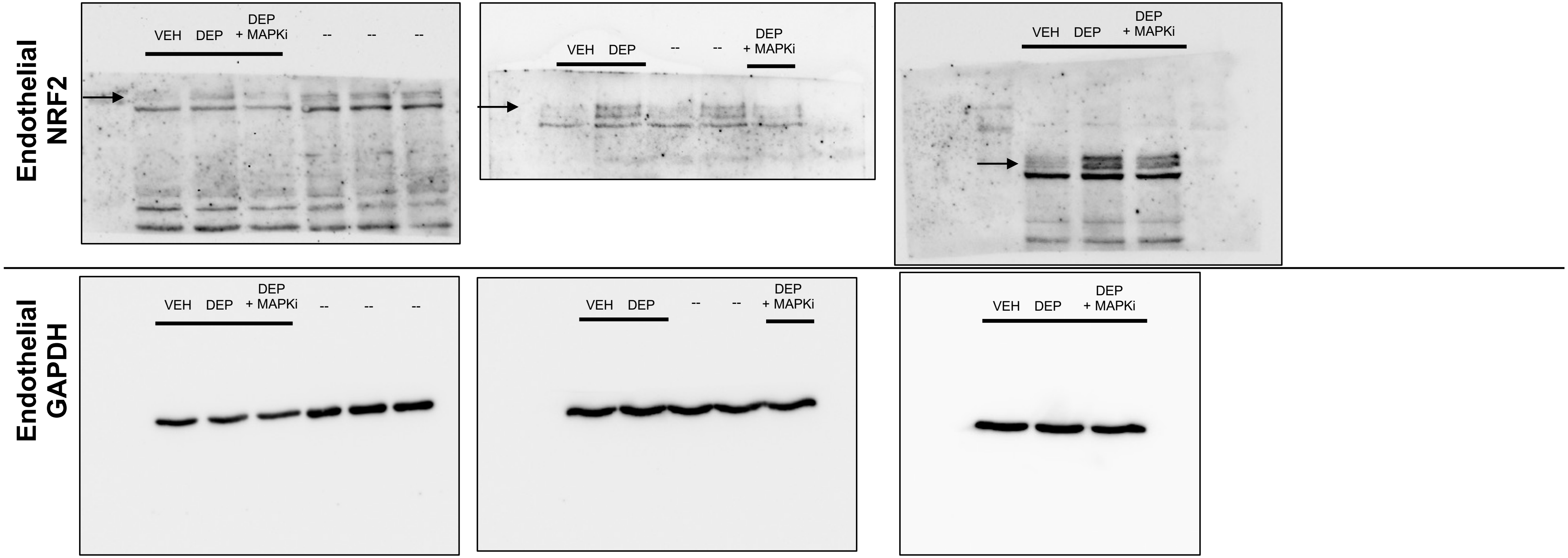

Endothelial NRF2
Endothelial GAPDH
VEH
DEP
DEP + MAPKi
--
VEH
DEP
--
--
DEP + MAPKi
VEH
DEP
DEP + MAPKi
