# Supplementary material for: Epithelial MAPK signaling directs endothelial NRF2 signaling and IL-8 secretion in a tri-culture model of the alveolar-microvascular interface following diesel exhaust particulate (DEP) exposure
Source: Part Fibre Toxicol. 2024 Mar 11;21:15. doi: 10.1186/s12989-024-00576-8 (PMC10926573; doi:10.1186/s12989-024-00576-8)
Supplement: Supplementary file 5 — Additional file 5. Method S1. CellROX Green HULEC Reactive Oxygen Species Detection. [file 12989_2024_576_MOESM5_ESM.docx]

**SUPPLEMENTAL METHODS**

***CellROX Green HULEC Reactive Oxygen Species Detection***

Cells were seeded in 12mm Transwell^®^ inserts as indicated in the ACRE Model Setup. On day 4 of the ACRE Model Setup, prior to exposure, seeded Transwell^®^ inserts were transferred to a temporary multi-well plate with pre-warmed (37 ˚C) HULEC exposure medium and returned to the humidified cell culture incubator. 500 µM CellROX Green solution was prepared in DMEM-F according to manufacturer’s protocol and spiked into the seeded HULEC wells. The plate was gently rocked to mix the CellROX Green solution and resulted in a final concentration of 5 µM. The HULECs were returned to a tissue culture incubator for 30 min to permit cellular uptake of the CellROX Green reagent. The HULEC medium containing excess CellROX reagent was then aspirated and replaced with pre-warmed (37 ˚C) CellROX Basolateral Exposure Medium (DMEM-F + 4 mM GlutaMAX with 1% FBS and 0.5% P/S). The apical medium was then aspirated from the ACRE inserts, the inserts were returned to the HULEC-seeded wells, and apical medium was replaced with either CellROX Apical Exposure Medium (Basal DMEM-F + 4 mM GlutaMAX with 0.5 uM dexamethasone and 0.5% penicillin/streptomycin) (VEH) or CellROX Exposure Medium containing DEP. Cell culture grade water was added to the void spaces of the multi-well plate and the plate was sealed with AeraSeal^TM^ film (Sigma-Aldrich, #A9224) to preserve humidity during the exposure in the plate reader. HULECs were then analyzed for fluorescence at excitation/emission 485/520 nm for 24 h on a CLARIOstar Plus plate reader (BMG LabTech) at 37 ˚C with 5% CO_2_ and ambient O_2_ levels. HULECs exposed to 20 µM menadione were used as a positive response control. The data shown represent the average (± SD) change in fluorescent signal from three independent experiments. The average change in fluorescent signal of each independent experiment was determined by subtracting the averaged first 10 min from the averaged last 10 min for three ACRE inserts (technical triplicates within an independent experiment per experimental condition). Statistical analysis was conducted in GraphPad Prism (version 9.3.1) using a paired, two-tailed *t*-test.
